# Trans-Reactivation: A New Epigenetic Phenomenon Underlying Transcriptional Reactivation of Silenced Genes

**DOI:** 10.1371/journal.pgen.1005444

**Published:** 2015-08-20

**Authors:** Maria Cristina Onorati, Walter Arancio, Vincenzo Cavalieri, Antonia M. R. Ingrassia, Giulio Pavesi, Davide F. V. Corona

**Affiliations:** 1 Dulbecco Telethon Institute, Palermo, Italy; 2 Università degli Studi di Palermo, Dipartimento STEBICEF–Sezione Biologia Cellulare, Palermo, Italy; 3 Department of Biosciences, University of Milano, Milano, Italy; Rutgers University, UNITED STATES

## Abstract

In order to study the role played by cellular RNA pools produced by homologous genomic loci in defining the transcriptional state of a silenced gene, we tested the effect of non-functional alleles of the *white* gene in the presence of a functional copy of *white*, silenced by heterochromatin. We found that non-functional alleles of *white*, unable to produce a coding transcript, could reactivate *in trans* the expression of a wild type copy of the same gene silenced by heterochromatin. This new epigenetic phenomenon of transcriptional *trans*-reactivation is heritable, relies on the presence of homologous RNA’s and is affected by mutations in genes involved in post-transcriptional gene silencing. Our data suggest a general new unexpected level of gene expression control mediated by homologous RNA molecules in the context of heterochromatic genes.

## Author Summary

We discovered a new epigenetic phenomenon we called *trans-*reactivation. We found that genes, unable to produce a functional coding transcript, but with the potential of transcribing other RNA’s within their gene body, strongly reactivate the transcription of a wildtype copy of the same gene silenced by heterochomatin. This new epigenetic phenomenon is heritable, relies on the presence of diffusible RNAs able to carry and transfer epigenetic information and is affected by mutations in genes involved in Post-Transcriptional Gene Silencing. Our data strongly suggest that homologous non-coding RNA can reactivate the expression of genes silenced by heterochromatin, thus defining a new unpredicted level of gene expression control in the context of heterochromatic genes.

## Introduction

In recent years it has become increasingly evident that the expression of eukaryotic genomes is far more complex than it had been previously explored. An emerging body of evidence, coming from next generation sequencing approaches, is showing that the genomes of all studied eukaryotes are almost entirely transcribed, generating an enormous number of non-coding RNAs (ncRNAs) [1]. The ENCODE project showed that at least 90% of analyzed eukaryote genome is transcribed in different cell types, indicating that there is a huge reservoir of RNA molecules with potentially unexplored biological function [2,3,4]. These RNAs are remarkably different in their number, size, subcellular localization, and mechanisms of action, and many are essential to finely control gene expression as well as genomic plasticity [5].

It is becoming increasingly clear that some ncRNAs are part of epigenetic regulatory networks with striking evolutionary conservation [6,7]. For example, dynamic changes in chromatin function are frequently transacted by nuclear RNA signaling pathways. Although the evolutionarily conservation and precise molecular mechanisms are poorly understood, a differential recruitment of a hierarchy of chromatin modifying complexes to specific loci by RNAs sets precise transcriptional states leading to differentiation [8,9,10,11,12]. Moreover, the unusual epigenetic phenomena of *paramutation* [13,14,15], *trans-induction* [16] and *transvection* [17,18] observed in a variety of higher eukaryotes involve the activity of ncRNAs that ‘rewrite’ the transcriptional state of an allele, in processes that apparently escape classic Mendel’s laws of genetic inheritance. These epigenetic phenomena are clear examples of ncRNA-directed regulatory processes that transfer epigenetic information both across cells, between tissues and across generations, though the mechanisms underlying these phenomena still remain elusive.

In order to unveil the role played by cellular RNA pools produced by homologous genomic loci in changing the transcriptional state of a silenced gene, we used classic Position Effect Variegation (PEV) assays in the model system *D*. *melanogaster* [19,20] and tested the effect of non-functional alleles of the *white* gene in the presence of a functional copy of *white*, silenced by heterochromatin (*w* ^*m4h*^). Surprisingly, we found that several non-functional *white* alleles, unable to produce the main wildtype *white* coding transcript but with the potential of transcribing other RNA’s from the *white* locus, *trans*-reactivate the variegating *w* ^*m4h*^ line thus increasing eye pigmentation. Strikingly, the presence of non-functional *white* alleles cause an increase in the *w* ^*m4h*^ gene transcript as well as an opening in the chromatin structure at the *w* ^*m4h*^ locus. Remarkably, this new epigenetic phenomenon is heritable, relies on the presence of diffusible homologous RNA’s, and is affected by mutations in genes involved in post-transcriptional gene silencing. Overall, our data strongly indicate that *trans*-reactivation is a new epigenetic phenomenon that positively control gene expression in the context of heterochromatin through homologous RNA molecules.

## Results

### Alleles of *white* can suppress *w* ^*m4h*^ eye color variegation

**Fig 1:**
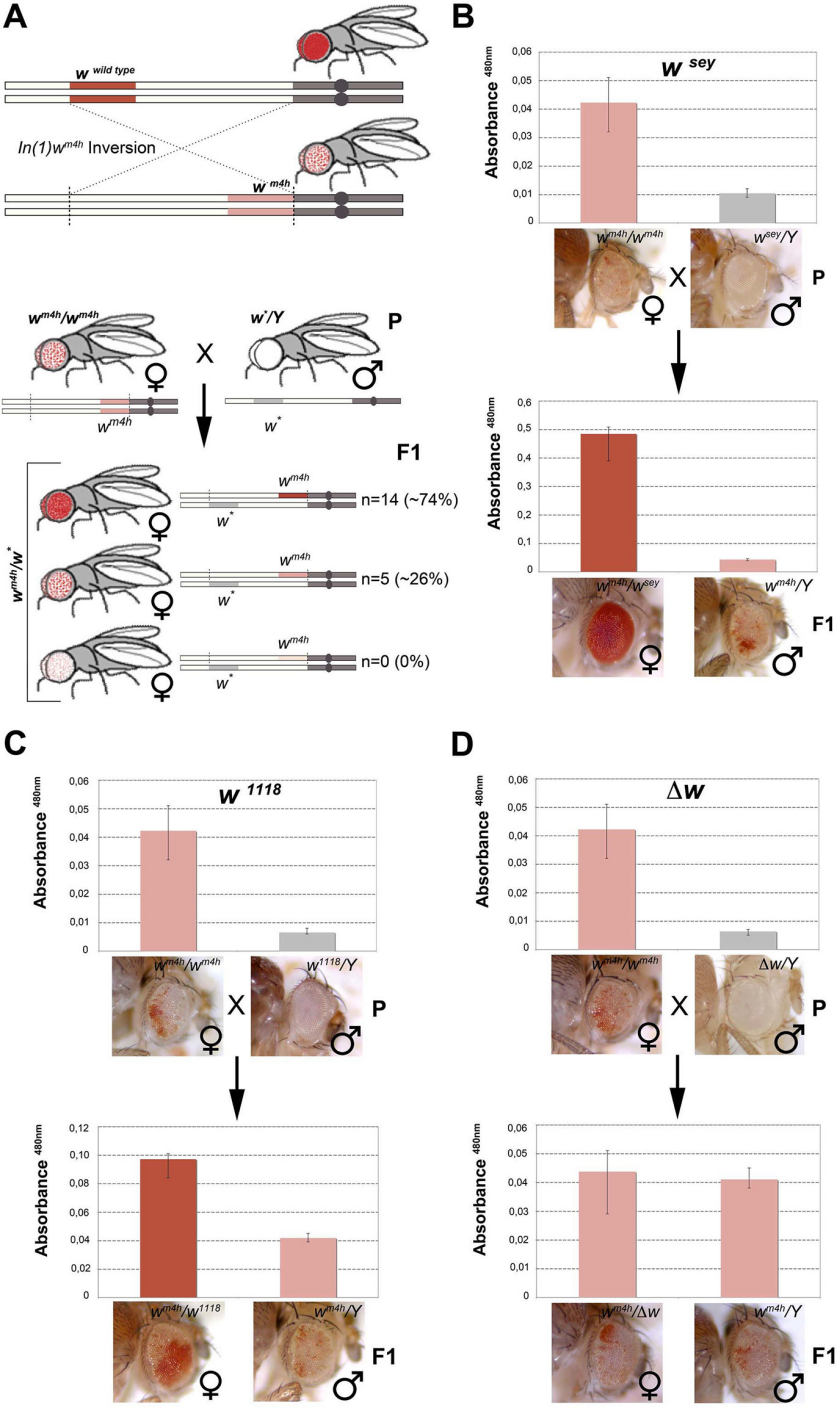
Hypomorphic and loss-of-function alleles of the *white* gene increase *w* ^*m4h*^ eye color pigmentation. **(A)** Upper panel; schematic representation of the *In(1)w* ^*m4h*^ inversion juxtaposing the *w* wildtype gene in the proximity of X chromosome pericentric heterochromatin, resulting in the generation of the *w* ^*m4h*^ allele with silenced *w* expression in most eye cells and leading to a variegated eye color. Lower panel; parental (P) variegating *w* ^*m4h*^ females were crossed with males carrying a hypomorphic or loss-of-function allele of the *white* gene (w*). The recovered progeny (F1) was scored for increased, decreased or unchanged eye color pigmentation. Number of alleles recovered (n) and corresponding percentage (%) of total alleles screened are reported. Eye pigment quantification of parental stocks (P) and of the resulting trans-heterozygous female (*w* ^*m4h*^ /w*) and control male (*w* ^*m4h*^ */Y*) progeny (F1) for the *w* ^*sey*^ **(B)**, *w* ^*1118*^ **(C)** alleles and *Δw* **(D)**, are shown together with representative eye pictures for each genotype tested. P and F1 eye pigment quantification graphs have different scale.

The *white* (*w*) gene encodes an ABC transporter essential for the red pigment transportation in the compound *Drosophila* eye. The *In(1)w* ^*m4h*^ X chromosome inversion places the fully functional wildtype euchromatic *w* gene adjacent to a region of pericentromeric heterochromatin, creating the variegated *white-mottled 4* (*w* ^*m4h*^) allele that is characterized by the random inactivation of *w* by the heterochromatin spreading from the inversion chromosome breakpoint (Fig 1A, upper panel). This cell autonomous transcriptional inactivation once established is clonally inherited, and it is responsible for an eye with a variegated expression of the red pigment, constituting an example of the genetic phenomenon known as Position Effect Variegation (PEV) [20].

Genetic screens have shown that a large number of mutations alter PEV phenotypes, resulting in the isolation of enhancers *E(var)s* or suppressors *Su(var)s* of variegating phenotypes. The vast majority of these modifiers were originally isolated in *Drosophila* as dominant mutations that suppressed or enhanced eye color variegation caused by the *w* ^*m4h*^ allele [21]. The molecular characterization of those mutants have shown that the products of *E(var)’s* and *Su(var)’s* are structural components of chromatin, or enzymes that covalently modify chromatin proteins [19]. However, to date the effect exerted by non-functional *w* alleles on the *w* ^*m4h*^ chromatin-dependent variegation is unknown. Therefore, we took advantage of the classic *w* ^*m4h*^ PEV assay to analyze if non-functional alleles of the *w* gene were able to modify eye color variegation when in trans-heterozygosis with the *w* ^*m4h*^ allele (Figs 1A, lower panel, and S1A and S1C).

We screened 19 loss-of-function or hypomorphic *w* alleles (w*) for genetic interaction with *w* ^*m4h*^, and measured eye red pigment to quantify the strength of the genetic interaction [22]. The pilot screen resulted in the isolation of 14 suppressors (74% of total w* alleles screened) able to increase *w* ^*m4h*^ eye color pigmentation (Fig 1A and S1 Table). Among the tested hypomorphic alleles that behaved as robust suppressors of *w* ^*m4h*^ variegation, the *w* ^*sey*^ allele interacted more strongly (Figs 1B and S2). Moreover, loss-of-function alleles of *w*, including the widely used *w* ^*1118*^ allele, showed a weak but highly reproducible suppression effect (Figs 1C and S3). Notably, F1 *w* ^*m4h*^ */Y* males derived from the crosses did not show any increase in eye color pigmentation (Fig 1B and 1C), indicating that the genetic locus responsible for the increased eye color pigmentation in the *w* ^*m4h*^ /w* trans-heterozygous females is carried by the w* bearing X chromosome. Reciprocal crosses using multiple *w* ^*m4h*^ balanced lines (S1B, S1D, S1E and S1F Fig) and classic recombination mapping (S1G and S1H Fig) confirmed the effect of the w* loci tested in increasing eye color pigmentation of *w* ^*m4h*^. Interestingly, deletions spanning the *w* locus (*Δw*) did not show any modification of the *w* ^*m4h*^ variegation (Fig 1D and S1 Table). Moreover, none of the w* alleles tested was able to reduce the red pigmentation of *w* ^*m4h*^, while 5 of them (26%) had no effect (Fig 1A, lower panel, and S1 Table). The pilot screen we conducted suggested that the presence of *white* genomic homologous sequences of non-functional w* alleles could reactivate in *trans*-heterozygosis the *w* ^*m4h*^ heterochromatic silenced locus.

### The suppression of *w* ^*m4h*^ variegation by w* alleles is a robust effect, unrelated to classic *Su(var)* function and independent from chromosome pairing

**Fig 2:**
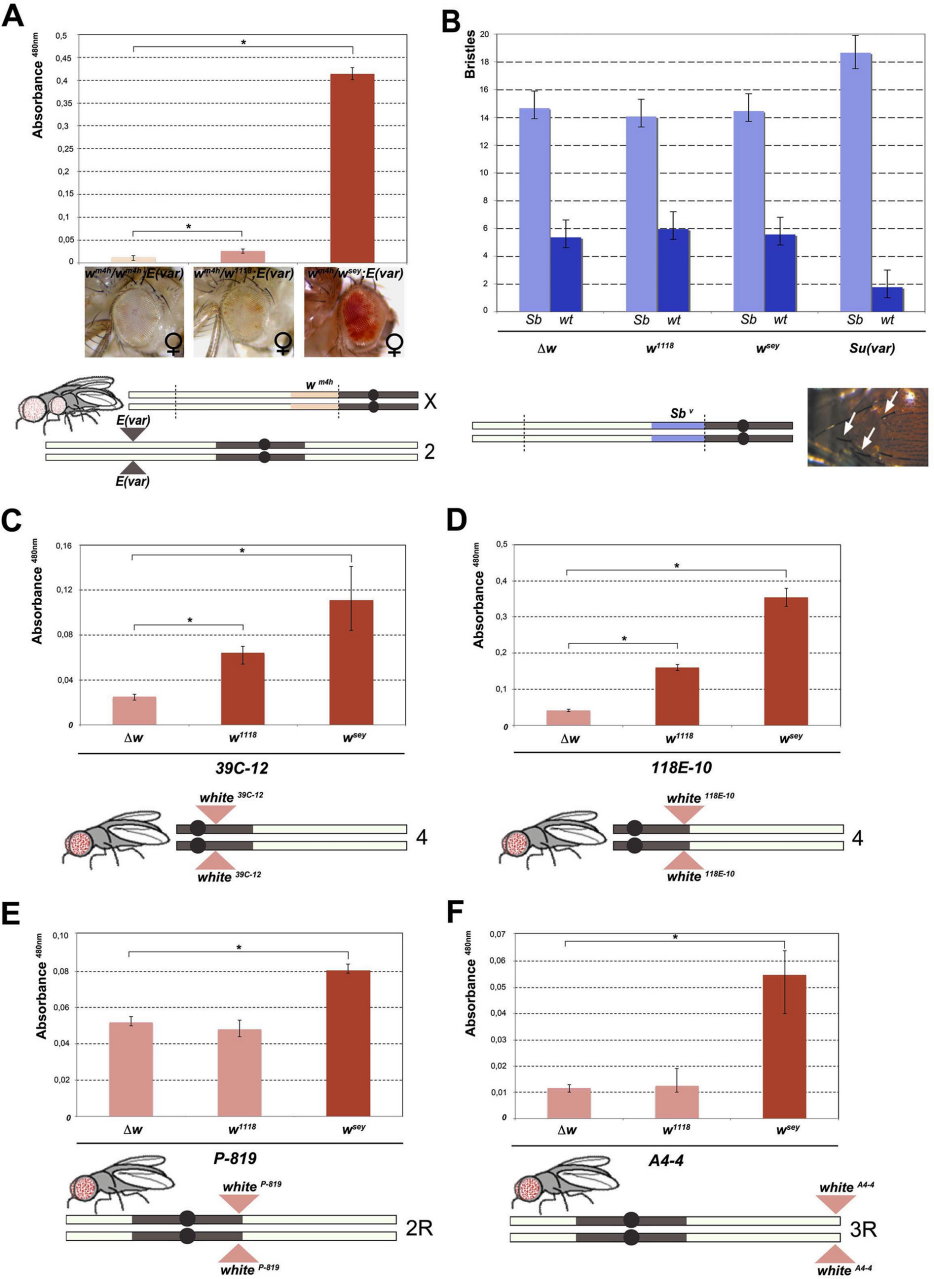
Effect of w* alleles on *E(var)*, *Sb* ^*v*^ and *w* variegating autosomal insertions. **(A)** Upper panel; eye pigment quantification of females *w* ^*sey*^, *w* ^*1118*^ and *Δw* trans-heterozygous with *w* ^*m4h*^, carrying one copy of the dominant *E(var)3−1* ^*01*^ mutation. Lower panel; schematic representation of the *E(var)3−1* ^*01*^; *w* ^*m4h*^ line used in the eye variegation assay. **(B)** Upper panel; quantification of the effect of *Δw*, *w* ^*1118*^, *w* ^*sey*^ and *Su(var)* ^*4–20*^ on the number of short (*Sb*) vs normal (*wt*) bristle length in the *Sb* ^*v*^ variegating line. Lower panel; representation of *T(2*:*3)Sb* ^*v*^ translocation. Activation of the dominant *Sb* allele results in short bristles (white arrows). **(C-E)** Upper panels; eye pigment quantification of *w* ^*sey*^, *w* ^*1118*^ and *Δw* males carrying one copy of the IV and II chromosome pericentric heterochromatin autosomal *w* variegating insertion 39C-12, 118E-10 and P-819. Lower panel; schematic representation of autosomal insertions **(F)** Upper panel; eye pigment quantification of *w* ^*sey*^, *w* ^*1118*^ and *Δw* males carrying one copy of the III chromosome telomeric heterochromatin *w* variegating insertion *A4-4*. Lower panel; schematic representation of autosomal insertions. The asterisks indicate the statistical significance (< 0.05) of differences in eye pigment using Student's *t*-test. For cross details see S4 Fig.

To further characterize the effect of *w* ^*sey*^ and *w* ^*1118*^ alleles (from now on used as representative examples of w* alleles giving a strong and weak interaction with *w* ^*m4h*^, respectively), we first decided to measure the strength of their suppression on the *w* ^*m4h*^ variegation. Interestingly, in the presence of a strong *E(var)* mutation encoding for a factor contributing to the opening of chromatin [23], both *w* ^*1118*^ and *w* ^*sey*^ retain their ability to increase eye pigmentation of *w* ^*m4h*^, though *w* ^*1118*^ with a weaker strength (Figs 2A and S4A). This data indicate that the suppressing w* alleles are able to increase eye pigmentation even when the *w* ^*m4h*^ allele is in a strongly silenced heterochromatin context.

Next we wanted to test the ability of w* alleles to behave as suppressors in other PEV assays, a feature that is shared by classic *Su(var)s* encoding for factors responsible for the establishment and maintenance of inactive chromatin [24]. We choose the *Sb* ^*v*^ PEV assay, characterized by the *T(2;3)Sb* ^*v*^ translocation that juxtaposes the dominant *Sb* mutation to centric heterochromatin, resulting in mosaic flies with both short (*Sb)* and normal bristles (Fig 2B). As expected, when the *Sb* ^*v*^ stock was crossed with a strong *Su(var)* [25] a significant suppression was observed in the frequency of *Sb* bristles (Figs 2B, upper graph, and S4B). However, both *w* ^*1118*^ and *w* ^*sey*^ as well as the *w* locus deletion (*Δw*) were not able to increase the number of short bristles when crossed with *Sb* ^*v*^. These results suggest that the reactivating w* alleles do not likely encode for a factor that, beyond its role in eye pigmentation, it is also responsible for chromatin structure organization. Thus, our data strongly indicate that the mechanism underlying *w* ^*m4h*^ suppression by w* alleles is very likely unrelated to classic *Su(var)’s* function.

The suppression effect of w* alleles we observed over *w* ^*m4h*^ is highly reminiscent of transvection, a well-studied epigenetic phenomenon that results from the interaction between an allele on one chromosome and the corresponding allele on the homologous chromosome. Transvection may lead to both gene activation or repression, and is strongly dependent upon chromosome pairing [18]. However, because of the large *In(1)w* ^*m4h*^ inversion, the *w* ^*m4h*^ bearing chromosome is never paired with the w* containing sister chromatid in the suppressed trans-heterozygous females (Fig 1A), thus suggesting that the effect we observed could not be assimilated to classic transvection. However, to exclude intra-chromosomal long distance pairing or X chromosome specific effects, we tested the ability of w* alleles to de-repress variegated wildtype *w* insertions in pericentric and telomeric autosomal chromosome heterochromatin [26,27,28]. In all lines tested the strong *w* ^*sey*^ and in some cases also the weak *w* ^*1118*^ allele, but not the *Δw* deletion, consistently increased eye pigmentation of variegated wildtype *w* pericentric or telomeric autosomal insertions (Figs 2C–2F and S4C). Our data strongly suggest that the increase in eye pigmentation exerted by w* alleles over *w* ^*m4h*^ is independent from the type of heterochromatin tested, it does not rely on intra-chromosomal pairing and it is chromosome independent.

### w* alleles can increase *w* ^*m4h*^ eye pigmentation by opening chromatin at the *w* ^*m4h*^ locus and increasing the levels of wild type *white* coding transcripts

**Fig 3:**
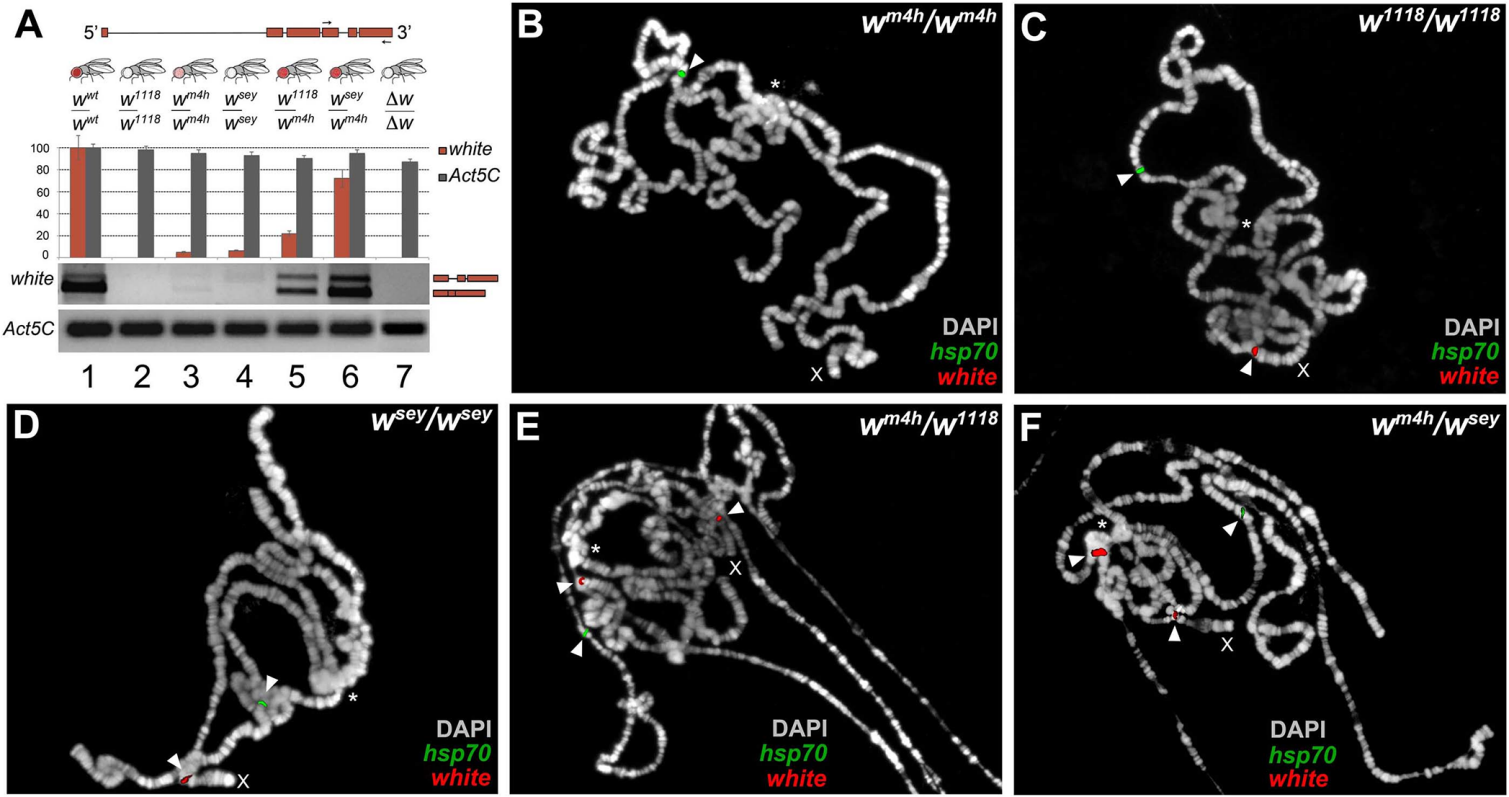
Increased levels of wildtype *white* coding transcripts and *w* ^*m4h*^ chromatin locus opening by w* alleles. **(A)** Structure of the *white* gene shows the 3’ region used to design the primers (arrows) for semi-quantitative RT-PCR conducted on total RNA, derived from adult heads dissected from the different genomic combinations shown. The graph shows the levels of amplified *w* transcripts. Primers designed against the *Act5C* transcript have been used as internal control. The two amplified bands represent unspliced and spliced forms of the *white* transcript. Polytene chromosome FISH using genomic probes covering the entire *w* gene (with the exception of the first intron) and coding sequences for the *hsp70* gene (mapping chromosome 3R) on homozygous *w* ^*m4h*^ */w* ^*m4h*^ **(B)**, *w* ^*1118*^ */w* ^*1118*^ **(C)**, *w* ^*sey*^ */w* ^*sey*^ **(D)** and trans-heterozygous *w* ^*m4h*^ */w* ^*1118*^ **(E)** and *w* ^*m4h*^ */w* ^*sey*^ **(F)** allelic combinations. FISH signals for *white* (red) and *hsp70* (green) genomic sequences are indicated by arrowheads. The asterisks indicate the region of pericentric heterochromatin. The X indicates the chromosome where the *w* gene maps. FISH signals for the *w* locus are detected in the *w* ^*1118*^ */w* ^*1118*^ and *w* ^*sey*^ */w* ^*sey*^ chromosomes because in these genotypes the *w* genomic sequences reside on euchromatic accessible region.

To explore the correlation of increased eye pigmentation in trans-heterozygous interacting females (*w* ^*m4h*^ /w*) and transcriptional activation of the *w* gene, we conducted semi-quantitative RT-PCR on total RNA extracted from adult heads. To measure the amount of full-length wildtype *white* coding mRNA we designed primers against the 3’ of the transcript that are able to amplify the fully transcribed spliced and unspliced mRNA products (Fig 3A). As expected, the RNA extracted from wildtype flies shows a robust amplification of *w*, while flies carrying the *w* ^*1118*^ loss-of-function allele or the *Δw* deletion do not show detectable levels of *white* transcripts (Fig 3A, compare lanes 1 with 2 and 7). Moreover, while the *w* ^*m4h*^ stock produced very low levels of wildtype coding *white* transcripts (Fig 3A, lane 3), the hypomorphic *w* ^*sey*^ flies produced only modest levels of unspliced *white* transcripts (Fig 3A, lane 4). Remarkably, when *w* ^*1118*^ or *w* ^*sey*^ are introduced *in trans* with *w* ^*m4h*^, we observed a highly reproducible and robust amplification of *white* transcripts (Fig 3, lanes 5 and 6). These data strongly correlate the levels of eye pigmentation, we observed in the trans-heterozygous w*/w ^*m4h*^ adults, with the levels of expressed coding *white* transcripts.

However, an increased *white* transcript stability in w*/w ^*m4h*^ trans-heterozygous could in theory also explain the observed high levels of amplification of coding *white* transcripts. Therefore, we tested the ability of mutants in genes known to be involved in maternal and zygotic fly mRNA stabilization for their ability to modify the levels of eye pigmentation normally scored in w*/w ^*m4h*^ adults. However, none of the mutants tested, including *pumilio* (*pum*) and *smaug* (*smg*), were able to significantly change eye pigmentation in our PEV assay (S5A Fig). These data strongly suggest that the increased levels of eye pigmentation observed in w*/w ^*m4h*^ trans-heterozygous females, cannot be directly correlated with general changes in *white* mRNA stability (S5A Fig).

While, for all the interacting loss-of-function w* alleles, we could be sure that any *white* coding transcript we detect could only be produced from the wildtype *w* ^*m4h*^ locus, the same conclusion cannot be drawn for the interacting hypomorphic w* alleles. Therefore, we decided to use genomic probes from the *white* genomic locus to conduct fluorescent in situ hybridization (FISH) analyses on polytene chromosomes, for monitoring the level of accessibility of the interacting w* and *w* ^*m4h*^ chromatin loci. Polytene chromosomes represent a special structural organization of *Drosophila* salivary glands, consisting of polyploid interphase nuclei, which originate by repeated rounds of DNA replication without cell division. *Drosophila* polytene chromosomes have proven to be an invaluable cytogenetic tool to examine chromosome structure. Moreover, DNA FISH probes hybridization is dependent on the level of replication of specific genomic regions on polytene chromosomes, which in turn depends on the overall level of transcriptional activity present on that locus [24].

Exploiting this special feature of polytene chromosomes, we designed genomic probes covering both the *w* locus and the coding sequences of the *hsp70* gene as internal positive control for FISH probe chromatin accessibility. As expected, *w* ^*m4h*^ homozygous female chromosomes gave a FISH signal for the *hsp70* gene but failed to show a detectable band from the *w* ^*m4h*^ pericentric chromatin region because this heterochromatic locus is under-replicated [24] (See arrowhead and asterisk in Fig 3B). On the other hand, homozygous chromosomes for the *w* ^*1118*^ and *w* ^*sey*^ alleles or wildtype flies gave detectable FISH signals, for both the *w* and *hsp70* loci (see arrowheads in Figs 3C and 3D and S5B). Remarkably, *w* ^*m4h*^ */w* ^*1118*^ and *w* ^*m4h*^ */w* ^*sey*^ but not *w* ^*m4h*^ */∆w* trans-heterozygous female chromosomes, on top of the expected signals coming from the *w* and *hsp70* loci, gave a highly reproducible FISH band originating from the pericentric *w* ^*m4h*^ chromatin locus (see arrowhead in the vicinity of the asterisk in Figs 3E and 3F and S5B). This analysis strongly suggests that FISH signals detected from the *w* ^*m4h*^ locus in trans-heterozygous w*/w ^*m4h*^ chromosomes are the result of increased DNA replication, consistent with an increase in transcription at the *w* ^*m4h*^ locus, as also found for other classic *Su(var)s* [24]. In conclusion, our data strongly indicate that the increase in eye pigmentation, observed in the *w* ^*m4h*^ /w* trans-heterozygous combinations, is strongly correlated with a reopening of wildtype heterochromatic *w* ^*m4h*^ locus by the presence of homologous genomic sequences present in the interacting loss of function and hypomorphic w* alleles. We called the ability of w* alleles to re-reactivate wild type *white* at the *w* ^*m4h*^ locus: *trans*-reactivation.

### *trans*-reactivation is heritable

**Fig 4:**
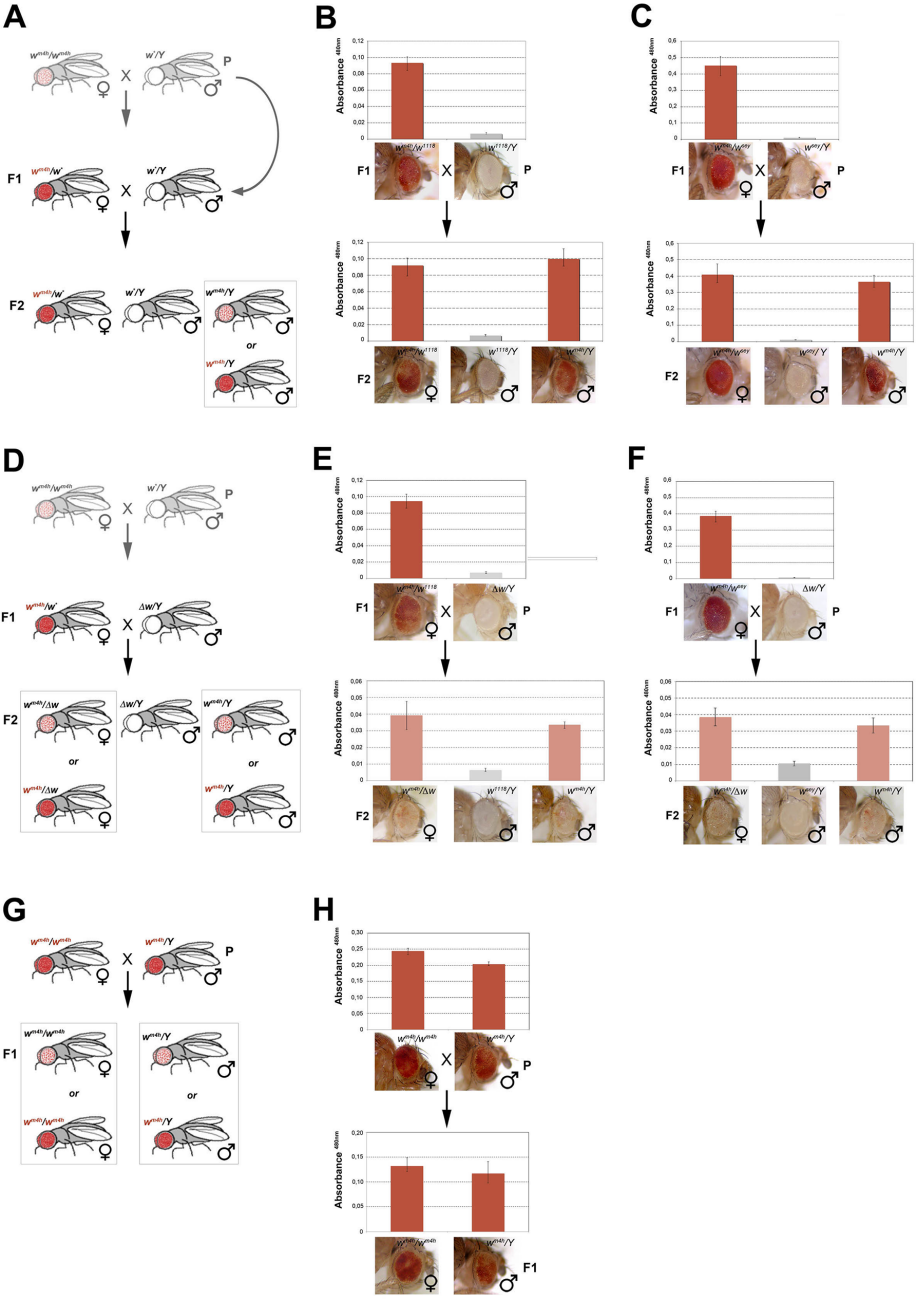
*trans*-reactivation of w* over *w* ^*m4h*^ is heritable. **(A)** Cross scheme used to test trans-generational inheritance of *w* ^*m4h*^ *trans*-reactivation. Parental (P) variegating *w* ^*m4h*^ */w* ^*m4h*^ homozygous females were crossed with males carrying the *trans*-reactivating w* allele. The (F1) *trans*-reactivated *w* ^*m4h*^ */w* ^*m4h*^ female was back crossed with w*/Y males carrying the *trans*-reactivating w* allele. Finally, the F2 progeny was scored for eye color pigmentation in control *w* ^*m4h*^/w* females, w*/Y males and the experimental *w* ^*m4h*^/*Y* males progeny, where the w* trans-reactivating allele had segregated. The possible F2 *w* ^*m4h*^/*Y* males eye phenotypes coming from the cross are boxed. Eye pigment quantification of the P and F1 stocks, together with the resulting F2 control trans-heterozygous female (*w* ^*m4h*^ /w*), control male (*w* ^*m4h*^ */Y*) and experimental segregating *w* ^*m4h*^ */Y* male progeny for the *w* ^*1118*^ **(B)** and *w* ^*sey*^ **(C)** alleles, together with representative eye pictures for each genotype tested are shown. **(D)** Cross scheme used to test if the *Δw/Y* males stock, carrying a deletion of the entire *w* locus, was able to induce the inheritance of *w* ^*m4h*^ *trans*-reactivation in F2 *w* ^*m4h*^ */Δw* females and *w* ^*m4h*^ */Y* male progeny derived from segregating *w* ^*m4h*^ */w* ^*1118*^ **(E)** and *w* ^*m4h*^ */w* ^*sey*^ **(F)** F1 trans-heterozygous. **(G)** Cross scheme showing the possible F1 progeny arising from an homozygous *trans*-reactivated *w* ^*m4h*^ */w* ^*m4h*^ female crossed with a *trans*-reactivated *w* ^*m4h*^/*Y* male, **(H)** and the corresponding eye pigment quantification of the P and F1 progeny obtained with crosses with stably *trans*-reactivated parental *w* ^*m4h*^ */w* ^*m4h*^ females and *w* ^*m4h*^/*Y* males (produced with the *trans*-reactivating *w* ^*sey*^ allele).

The epigenetic interaction between the w* and *w* ^*m4h*^ alleles, leading to an increase in *white* transcription as a consequence of chromatin reopening at the silenced *w* ^*m4h*^ locus, is a phenomenon highly reminiscent of the epigenetic switch occurring in paramutation. In this epigenetic phenomenon, identified in plants, mice, and recently also in flies, the paramutating allele has the ability to change the activity state of its partner allele on the homologous chromosome (usually to a silent state) [14,15,29]. This effect is dependent on homologous sequences present in the two interacting alleles, is heritable and once established, is independent from the presence of the paramutating allele. To investigate if the reactivation of the *w* ^*m4h*^ locus was heritable through meiosis, we crossed F1 *trans*-reactivated *w* ^*m4h*^/w* females with w*/Y males to look for trans-generational inheritance of *trans*-reactivation in the F2 progeny (Figs 4A and S6A).

In the F2 progeny we expect to score *w* ^*m4h*^/w* females (that should continue to show increased levels of eye pigmentation), *w* ^*^ */Y* males (deficient of red eye pigment), and finally *w* ^*m4h*^/*Y* males where the F1 *trans*-reactivated *w* ^*m4h*^ allele, inherited from the F1 mothers has segregated from the w* trans-reactivating allele. If F2 *w* ^*m4h*^/*Y* males have normal variegated eyes, the F1 *trans*-reactivated *w* ^*m4h*^ allele had lost its *trans*-reactivating potential during meiosis. On the other hand, if F2 *w* ^*m4h*^/*Y* males showed increased eye pigmentation, this would be a strong indication that the *trans*-reactivated state of the F1 *w* ^*m4h*^ allele is transmitted in the germline and inherited in the F2 generation (Fig 4A).

Remarkably, both *w* ^*1118*^ and *w* ^*sey*^ *trans*-reactivating alleles when segregated away from the F1 activated *w* ^*m4h*^ allele were able to generate F2 *w* ^*m4h*^/*Y* males with strong eye pigmentation (Fig 4B and 4C). However, when the same F1 trans-heterozygous *w* ^*m4h*^/w* females were crossed with *Δw /Y* males not bearing any homologous genomic *w* sequence (Figs 4D and S6B), the resulting F2 *w* ^*m4h*^/*Y* males and *w* ^*m4h*^/*Δw* females failed to *trans*-reactivate showing levels of eye variegation indistinguishable from parental (P) *w* ^*m4h*^ stocks (Fig 4E and 4F). Remarkably, when wild type *w+* carrying flies are tested for their ability to *trans*-reactivate in F2 *w* ^*m4h*^ males, we did not observe any *trans*-reactivation (S6C Fig). However, when we crossed homozygous *trans*-reactivated *w* ^*m4h*^ */w* ^*m4h*^ female with *trans*-reactivated *w* ^*m4h*^/*Y* male, where the *trans*-reactivating w* allele was no longer present in both lines, we could reproducible score only *trans*-reactivated progeny up to the F5 (Fig 4G and 4H).

Although, *trans*-reactivation also works in reciprocal crosses where the *trans*-reactivating w* allele is carried by the mother (S1E and S1F Fig), our data show that *w* ^*m4h*^ *trans*-reactivation is dependent upon the presence of non-functional w* homologous genomic sequences present in at least one gamete (the sperm, in this set of experiment). Moreover, since fully functional coding *wild type w* ^*+*^ alleles are unable to *trans*-reactivate, our data also indicate that the w* reactivating alleles likely encode *trans*-reactivating factors able to maintain the *trans*-reactivated state of *w* ^*m4h*^ coming from the other gamete, a feature that does not meet the classic definition of paramutation. Indeed, paramutated alleles maintain their state independently from the presence of the paramutating allele. However, crosses in which both parents have exclusively *w* ^*m4h*^ *trans*-reactivated alleles strongly maintain the *trans*-generational inheritance of *trans*-reactivated *w* ^*m4h*^, making *trans*-reactivation a new epigenetic phenomenon, distinct from paramutation, causing the trans-generational inheritance of *trans*-reactivated *w* ^*m4h*^.

### A diffusible factor carrying genetic information is responsible for *trans*-reactivation

A long-standing question in the understanding of the mechanisms underlying epigenetic memory inheritance is whether a genetic information could be transferred from one allele to another through physical pairing of homologous chromosomes, or via a diffusible intermediate carrying genetic information. Although, our data indicate that *trans*-reactivation of w* over *w* ^*m4h*^ is independent from classic chromosome pairing (Fig 2C–2F), some of the features of w* trans-reactivation are highly reminiscent of the process known as *trans*-inactivation observed for the *brown* (*bw*) locus in flies, and of *trans*-induction occurring at the globin gene cluster in mammals, two epigenetic phenomena opposite in their final transcriptional outcome, but highly dependent on long-range chromosome physical interaction [16,30].

Indeed, in *trans*-inactivation when two *bw* alleles are paired, heterochromatin insertion in one causes both alleles to associate with the heterochromatic centromere, suppressing *brown* expression [30]. On the other hand, in *trans*-induction intergenic transcription of the globin cluster specific for the erythroid cells can be induced in non-erythroid cells by the expression of transiently transfected globin genes [16]. Physical interaction of homologous chromatin region of the affected alleles appears to inactivate, in the case of *trans*-inactivation, or activate, in the case of *trans*-induction, chromosomal transcription. Indeed, the activating effect of w* alleles over *w* ^*m4h*^ could be explained by the two homologous inverted loci engaging on a long-range physical interaction. Under this model, the silent *w* ^*m4h*^ locus could dilute out heterochromatic factors over the non-functional w* homologous genomic sequences, causing a partial de-repression of *w* ^*m4h*^, thus explaining the opening of the chromatin locus, as well as the increased *white* transcription and eye pigmentation.

**Fig 5:**
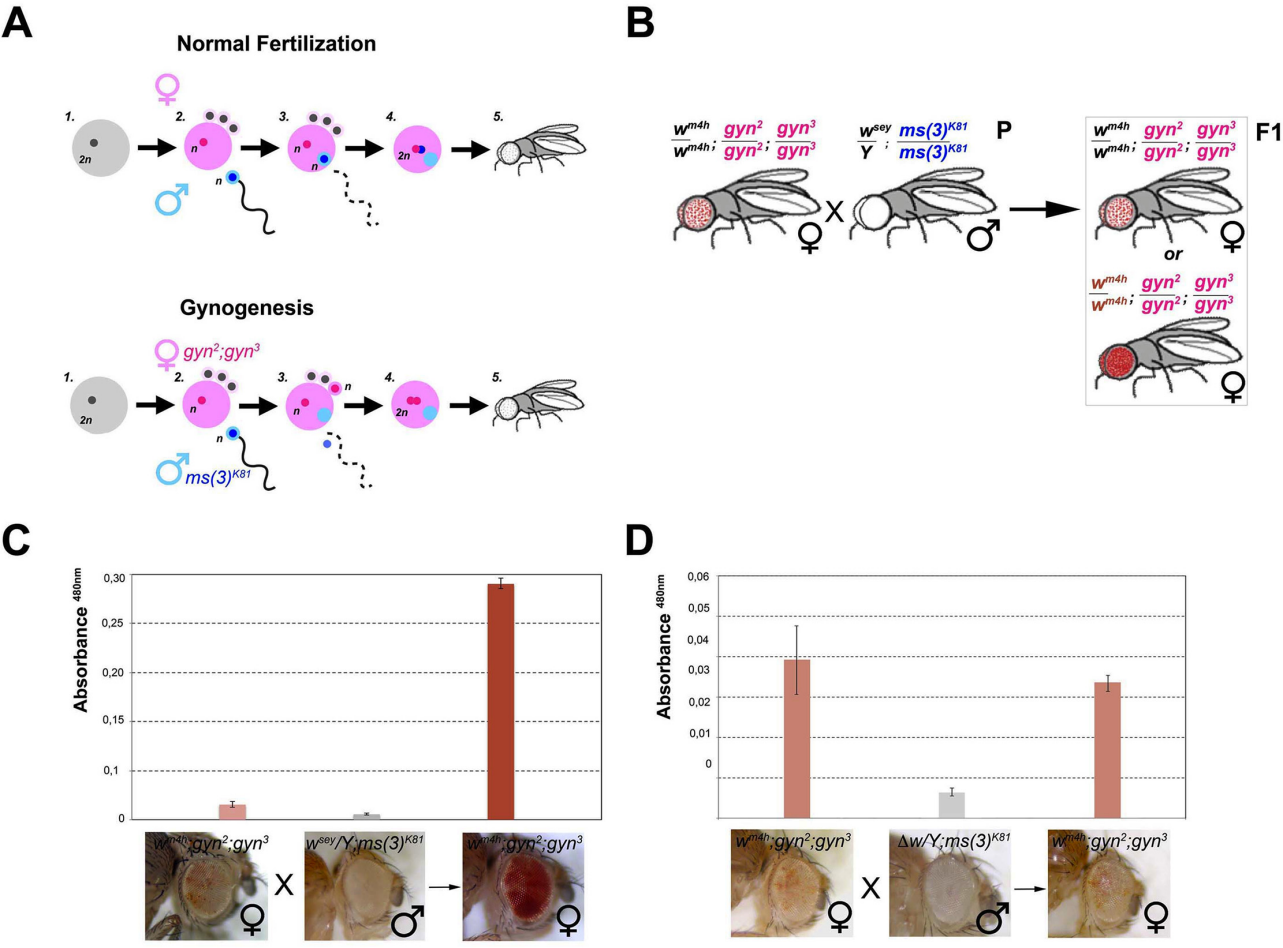
*trans*-reactivation is mediated by a diffusible factor. **(A)** Upper panel; schematic representation of normal fertilization leading to the generation of an adult fly. Lower panel; description of the process of gynogenesis in which a viable adult fly that does not carry any genetic contribution from the father is generated through the fertilization of a gynogenetic *gyn* ^*2*^, *gyn* ^*3*^ egg (where diploidy is restored by the fusion of two non-sister nuclei out of the four egg pronuclei which result from the second meiotic division) by a *ms(3)* ^*K81*^ sperm (one of a rare class of fly male sterile mutations in which sterility is caused by the elimination of male pronucleus after sperm entry into the egg). Despite, the male pronucleus (blue) does not contribute to the zygote, the male nucleoplasm (light blue) contributes together with the two female pronuclei (pink) to the development of a gynogenetic adult fly. **(B)** Cross scheme used to test if nucleoplasmic factors devoid of any male genomic contribution coming from the w* trans-reactivating allele can *trans*-reactivate *w* ^*m4h*^ in female gynogenetic adult flies. Possible F1 eye phenotypes coming from the cross are boxed. Eye pigment measurements of parental and gynogenetic progeny were conducted to score the presence of a *trans*-reactivated *w* ^*m4h*^ in **(C)** *w* ^*m4h*^ *; gyn* ^*2*^, *gyn* ^*3*^ female, *w* ^*sey*^ *; ms(3)* ^*K81*^ male parental stocks, and F1 gynogenetic *w* ^*m4h*^; *gyn* ^*2*^, *gyn* ^*3*^ progeny, or in control **(D)** *w* ^*m4h*^ *; gyn* ^*2*^, *gyn* ^*3*^ female, *Δw; ms(3)* ^*K81*^ male parental stocks, and F1 gynogenetic *w* ^*m4h*^; *gyn* ^*2*^, *gyn* ^*3*^ progeny. Eye pigment quantification graphs have a different scale.

In order to determine if w* trans-reactivation is mediated by a long range physical interaction between the homologous w* and *w* ^*m4h*^ loci or instead through diffusible factors produced by the homologous genomic sequences present in the w* alleles, we made use of the unique reproductive features of flies able to generate adults through the process of gynogenesis [31,32]. Gynogenesis is like parthenogenesis in that diploid zygotes inheriting all chromosomes from their mother can develop without a genetic contribution from fathers. However, even though gynogenetic diploid eggs do not require paternal chromosomes, they do require the physical penetration of the sperm into the mature egg and the contribution of paternal diffusible nuclear and cytoplasmic factors in order to initiate zygotic development (Fig 5A). In particular, when crossing the paternal effect lethal *ms(3)* ^*K81*^ males with gynogenetic *gyn* ^*2*^, *gyn* ^*3*^ females, gynogenesis occurs and the only possible progeny resulting from this cross are *gyn* ^*2*^, *gyn* ^*3*^ females (Fig 5A).

Therefore, we asked what happened to eye color pigmentation in the progeny resulting from *w* ^*m4h*^ gynogenetic *gyn* ^*2*^ *; gyn* ^*3*^ eggs fertilized by *ms(3)* ^*K81*^ males carrying the strong *trans*-reactivating *w* ^*sey*^ allele (Fig 5B). If a long range physical interaction between the male-carrying *w* ^*sey*^ and female-carrying *w* ^*m4h*^ chromosomes is required for *trans*-reactivation we should observe no increase in eye pigmentation in the *w* ^*m4h*^ *; gyn* ^*2*^ *; gyn* ^*3*^ F1 female progeny, because the father chromosomes do not contribute to the gynogenetic zygote (Fig 5B). However, if diffusible factors carrying epigenetic information are present in the *ms(3)* ^*K81*^ males encoding the *trans*-reactivating *w* ^*sey*^ allele, then diploid *w* ^*m4h*^ *; gyn* ^*2*^ *; gyn* ^*3*^ F1 female progeny should show *trans*-reactivated eyes (Fig 5B). Remarkably, F1 *w* ^*m4h*^ *; gyn* ^*2*^ *; gyn* ^*3*^ females, not carrying any genomic contribution from the father, show strong *trans*-reactivated eyes when fertilized by *ms(3)* ^*K81*^ males carrying the *trans*-reactivating *w* ^*sey*^ allele (Figs 5C and S7A). However, no effect on eye pigmentation was observed in the F1 progeny of gynogenetic females crossed with *ms(3)* ^*K81*^ males carrying the *Δw* deletion (Figs 5D and S7B).

The data resulting from the gynogenesis experiment strongly rule out the possibility that *trans*-reactivation is due to any type of long-range physical genomic interactions between the *w* ^*m4h*^ and w* alleles. Instead, our data strongly indicates that diffusible factors, present in the gamete carrying the homologous *trans*-reactivating w* allele, are responsible for the phenomenon of *trans*-reactivation of *w* ^*m4h*^.

### Mutations in genes encoding for factors involved in the generation, modification, and processing of ncRNA influence *trans*-reactivation

The diffusible factors able to mediate *trans*-reactivation should carry a genetic information coming from the w* allele, epigenetically transfer this information to the *w* ^*m4h*^ locus, and amplify this information in the zygote in order to exert their effect later in the fully differentiated adult eye tissue. The high diffusibility, self-amplification property, and capacity to epigenetically transfer information make RNA a great candidate for such a *trans-*reactivating diffusible factor. We therefore looked at the effect of known mutations in genes involved in the generation, modification and processing of a variety of family of small ncRNA in the process of *trans*-reactivation (S7C and S7D Fig).

**Fig 6:**
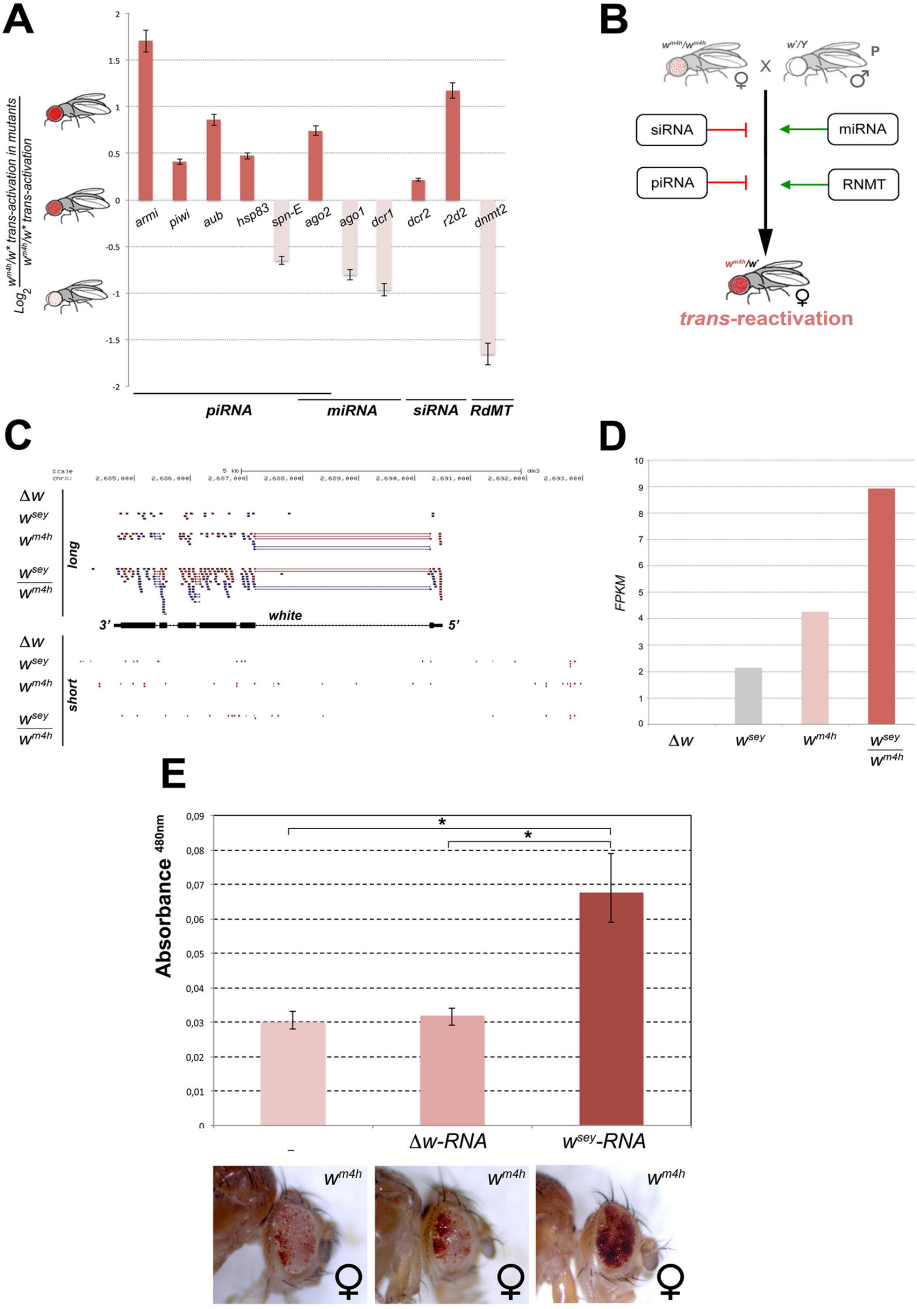
Mutations in genes encoding for factors involved in PTGS and RNA methylation influence *trans*-reactivation. **(A)** Loss of function mutations in *armitage* (*armi* ^Δ99^), *piwi* (*piwi* ^*06843*^), *aubergine* (*aub* ^*QC42*^), *hsp83* (*hsp83* ^*scratch*^), *spn-E* (*spn-E* ^*1*^), *argonaute2* (*ago2*), *argonaute1* (*ago1* ^*04845*^), *dicer1* (*dcr1*), *dicer2* (*dcr2*), *r2d2* (*r2d2* ^*1*^), and *dnmt2* (*dnmt2*), were tested in heterozygosis for their ability to modify the levels of eye pigmentation we scored in *w* ^*m4h*^/w* trans-reactivation. The Log_2_ ratio of *w* ^*m4h*^/w* trans-reactivation in the mutant background over the normal levels of *w* ^*m4h*^/w* trans-reactivation was calculated and plotted (see also S7D Fig). A positive value indicates that the mutation enhances *trans-reactivation* (dark orange bars), while a negative value is indicative of a suppression of *trans-reactivation* (pale orange bars). The strong *trans-reactivating w* ^*sey*^ allele was used in this assay. **(B)** A schematic representation of the biological significance of the data obtained shows that mutations in genes involved in the biogenesis of siRNA and piRNA counteract *trans*-reactivation (red bars). On the other hand, mutations in genes involved in the processing of miRNA and in RNA methylation (RdMT; RNA-dependent Methyl Transferase) promote *trans*-reactivation (green arrows). **(C)** High throughput RNA-Seq reads mapped on the *Drosophila* genome at the *white* gene locus for long and short RNA species purified from Malpighian tubules of *Δw*, *w* ^*sey*^, *w* ^*m4h*^ and *w* ^*sey*^ */w* ^*m4h*^ lines. **(D)** FPKM values plot obtained for the overall amount of long RNA species mapped at the *w* gene in the *Δw*, *w* ^*sey*^, *w* ^*m4h*^ and *w* ^*sey*^ */w* ^*m4h*^ lines. **(E)** Eye pigment quantification of *w* ^*m4h*^ females resulting from the injection of *w* ^*m4h*^ eggs with buffer (-) or total RNA extracted from testis of either Δw or *w* ^*sey*^ *Drosophila* males. The asterisks indicate the statistical significance (< 0.05) of differences in eye pigment using Student's *t*-test.

With the single exception of *spd-E* allele, mutations in genes encoding for factors involved in piRNA (*armi*, *piwi*, *aub*, *hsp83*, and *ago2*) and siRNA (*dcr2* and *r2d2*) biogenesis enhanced the *trans*-reactivating effect of w* over *w* ^*m4h*^ (Fig 6A). On the other hand, mutations in genes encoding factors involved in the production of miRNA (*ago1* and *dcr1*) suppress *trans*-reactivation, with the exception of *ago2* that is also involved in the piRNA pathway (Fig 6A). Recently, a non-coding RNA expressed from a human pseudogene was reported to regulate the corresponding protein-coding mRNA by acting as a decoy for microRNAs [33]. This study raised the questions about the potential ability of non-coding transcripts to act as ‘sponges’ to attract miRNAs, thus boosting the expression of miRNA target genes. If the *trans*-reactivating w* alleles produced miRNAs sponge RNA’s we should expect that mutations in genes affecting miRNA biogenesis should enhance the effect of the *trans*-reactivating w* alleles over *w* ^*m4h*^. However, our data clearly show that mutations in components of the miRNA processing machinery suppress *trans*-reactivation (Fig 6A and 6B), making it very unlikely that *trans*-reactivation could be explained by a miRNAs sponge effect. Remarkably, mutation in *dnmt2*, an RNA-dependent Methyl Transferase (RdMT) very recently shown to be involved in the phenomenon of paramutation in mice [34,35], has also strong suppressing effect on *trans*-reactivation (Fig 6A). Overall, our genetic interaction data strongly indicate that altering the biogenesis, the post-translational modification and processing of a variety of small ncRNA families interfere at various levels the efficiency of *trans*-reactivation (Fig 6B), strongly supporting the involvement of RNA molecules in the onset and maintenance of this new epigenetic phenomenon.

In order to identify unique specific RNA species produced by the w* alleles during the process of *trans*-reactivation of *w* ^*m4h*^ we conducted high throughput RNA sequencing analysis of total as well as small RNA enriched fractions from larval Malpighian tubules, an abundant fly larval tissue that express high levels of the *w* gene. However, the analysis of short RNAs did not result in the identification of specific or enriched RNA species in the *trans*-reactivated *w* ^*sey*^ */w* ^*m4h*^ line when compared to the *w* ^*sey*^ or *w* ^*m4h*^ lines alone (lower panel Fig 6C). On the other hand, the analysis of long RNA species clearly showed that the *trans*-reactivated *w* ^*sey*^ */w* ^*m4h*^ line overall produces more coding RNA from the *w* locus than the single *w* ^*sey*^ or *w* ^*m4h*^ lines alone (upper panel Fig 6C and 6D), in line with our previous semi quantitative RT-PCR analysis (Fig 3A). In conclusion, our RNA sequencing data did not allow us to identify specific or discrete RNA species present in the *w* ^*sey*^ *trans*-reactivating allele that could explain the onset of *trans*-reactivation.

However, if RNA species produced at the homologous w* alleles could influence *in trans* the transcription of the silenced *w* ^*m4h*^ locus, we expect that injection of *w* ^*m4h*^ eggs with total RNA extracted from w* individuals should result in *trans*-reactivated adult flies. Remarkably, when we injected *w* ^*m4h*^ eggs with total RNA extracted from *w* ^*sey*^ testis (Fig 6E) we obtained adults with *trans*-reactivated eyes. However, *w* ^*m4h*^ eggs injected with RNA derived from *Δw* testis had no effect (Fig 6E), strongly indicating that RNA molecules produced by the homologous *w* ^*sey*^ allele are responsible for the *trans*-reactivation of the *w* ^*m4h*^ locus. In conclusion, we propose that the RNA produced by the w* alleles from the *w* locus can induce *in trans* the activation of the silenced *w* ^*m4h*^ locus through a yet to be defined mechanism in which post transcriptional gene silencing and RNA methylation factors are involved.

## Discussion

### Role for ncRNA in transcriptional regulation

Over the last decade we have come to appreciate that the eukaryotic chromatin is full of coding and non-coding overlapping transcripts, and that a lot of the genetic information is transacted by coding and non-coding RNAs. Indeed, the vast majority of genomes of all metazoans are transcribed into complex patterns of ncRNAs. Recent observations strongly suggest that ncRNAs contribute to the complex transcriptional networks needed to regulate cell function [36,37]. However, despite a rich variety of mechanisms by which RNA acts by repressing transcription have been identified, fewer studies have explored the role of RNA in the positively regulation of transcription. RNA is an integral component of chromatin [38] and many transcription factors and chromatin modifying enzymes have the capacity to bind RNA or complexes containing RNA or RNA-binding proteins [8]. Moreover, RNA-directed processes help to establish chromatin architecture and epigenetic memory [10]. Indeed, ncRNAs can act locally to regulate the epigenetic state of the nearby chromatin, often recruiting either repressing or activating chromatin remodeling complexes [8]. Given the high degree of sequence and locus specificity information, RNA could recruit chromatin remodelers and modifiers in a sequence-specific manner. Indeed, recent observations are beginning to reveal that RNA molecules can stimulate gene transcription. These RNA activators employ a wide array of mechanisms to up-regulate transcription of target genes, often functioning as DNA-tethered activation domains, as coactivators or modulators of general transcriptional machinery [39].

### *trans*-reactivation: A new level of gene expression control of silenced genes?

In search for a possible role played by cellular RNA pools produced by homologous genomic loci in changing the transcriptional state of a silenced gene, we found that non-functional alleles of the classic fly marker *white* could *trans-reactivate* the expression of wild type copy of *white* silenced by heterochromatin. We called this new epigenetic phenomenon *trans*-reactivation. This process is heritable over many generations, it relies on the presence of diffusible RNAs, and it is affected by mutations in genes involved in post-transcriptional gene silencing. Our data strongly suggest that the presence of a gene that does not produce a full coding transcript but only spurious transcription could *trans*-reactivate the expression of a functional copy of the same gene silenced by heterochromatin, defining a new unpredicted level of gene expression control in the context of heterochromatic genes.

### The mechanism underlying *trans*-reactivation

The mechanisms of action and many features of *trans*-reactivation appear to be different from the previously characterized epigenetic reprogramming events occurring in *paramutation*, *transinduction* and *transvection*. In *trans*-reactivation genomic regions, transcribing RNAs unable to produce a full coding transcript, can positively influence the transcriptional state of an homologous locus silenced by heterochromatin. However, how can the information present in the non-functional RNA molecules be ‘read’ and telecasted to specific silenced homologous genomic regions? RNA molecules have the potential to pair with other RNA molecule or with DNA. Possibly through their ability to form paired structures, RNA may function as a sensor and/or a messenger of homology. RNA can form duplexes with sequence homology with genomic promoter sequences thus promoting transcription [40]. Indeed, there is a great deal of evidence that enhancers and other promoter regulatory sequences are transcribed in the cell in which they are active, and that enhancer transcription is often needed to activate the transcription on their target gene [41]. Moreover, given that mutations in genes encoding for factors involved in posttranscriptional gene silencing modulate *trans*-reactivation, it remains to be determined whether some sequence specific small ncRNAs, that we failed to identify, may explain the mechanism of *trans*-reactivation. Furthermore, we do not know why some non-functional w* null or hypomorphic alleles failed to *trans*-reactivate their homologous silenced locus. Indeed, differences in *trans*-reactivation ability between different w* alleles will need future investigation, and may provide key information to understand the mechanism of action of *trans*-reactivation. However, independently from the mechanistic details of *trans*-reactivation, we are convinced that we identified a new epigenetic phenomenon of allelic communication that acts through diffusible RNA molecules. Nevertheless, much work need to be done to study the evolutionary conservation and the general ability of *trans*-reactivating RNAs in resetting the epigenetic and transcriptional state of homologous genetic loci.

## Materials and Methods

### *Drosophila* stocks and genetic crosses

Flies were raised and crossed at 20° on K12 Medium (US Biological). Unless otherwise stated, strains were obtained from Bloomington, Szeged, and DGRC Stock Centers. Recombination mapping of the *trans*-reactivating effect of w* alleles is described in S1 Fig. The *white* alleles used for the *trans*-reactivation assays are listed in S1 Table. The *w* ^*1*^ *; gyn-2* ^*1*^ *;gyn-3* ^*1*^ and *ms(3)K81* ^*1*^ */TM3*, *Sb* ^*1*^, *Ser* ^*1*^ lines were a generous gift from Dr. Mia Levine. The *w* ^*m4h*^; the *CyORoi/Sco—w* ^*m4h*^ *; PrDr/TM3 Sb*,*Ser—E(var)3−1* ^*01*^ *-* lines were donated by Dr. Gunter Reuter. The *39C12* and *118E-10* variegating autosomal lines were a gift from Dr. Sarah Elgin. The *cn1 P{PZ}AGO* ^*104845*^ */CyO—ry506*, *r2d2* ^*1*^ */CyGFP—aub* ^*HN2*^ */CyO—y1 w67c23;P{EPgy2}AGO2* ^*EY04479*^ */ Cy—Armi/TM3 ser*, *GFP—ru* ^*1*^ *st* ^*1*^ *spn-E* ^*1*^ *e* ^*1*^ *ca* ^*1*^ */TM3*, *Sb* ^*1*^ *Ser* ^*1*^ *—Dcr* ^*1*^ */TM6c*—*Hsp83* ^*Scratch*^ */TM3 Sb*,*Ser* and *T(2;3)Sb* ^*V*^ *⁄ TM3*,*Ser* lines were obtained from Dr. Maria Pia Bozzetti and Dr. Laura Fanti. The *Ago2* ^*null*^, *Dcr2* ^*null*^ */CyO*, *piwi* ^*06843*^ */CyO*, *aub* ^*QC42*^ */CyO* lines derive from Dr. Valerio Orlando Lab and the *P-819 and A4-4* lines from Dr. Stephane Ronsseray lab. Dr Frank Lyko provided the *Dnmt2* ^*Δ99*^ */CyO* line.

### Pigment and variegation assays

The eye pigment assay was performed in triplicate for each of the three biological replicate we conducted, as previously described [22]. Eyes from representative individuals of the genotype tested were photographed with an Olympus SZ61 stereomicroscope. For the dominant Stubble variegation (*Sb* ^*V*^) assay ten pairs of major dorsal bristles were analysed in 20 flies. The extent of *Sb* variegation was represented as the mean of *Sb* and WT bristles per genotype tested in three biological replicate.

### Fluorescence in situ hybridization (FISH)

Cytological preparations and FISH analysis were carried out as previously described [42] The P(CaSpeRA) plasmid containing the genomic sequences of the mini *white* as well as the *hsp70* genes was used as a template for generating the FISH probe. FISH signals were acquired with a Leica DM 4000B microscope (Leica).

### Semi-quantitative RT-PCR

Total RNA was purified from 20 heads of adult flies of the desired genotype, using TRIzol reagent following the manufacturer’s (Invitrogen) recommended protocol. First strand cDNA was synthesized using MuLV reverse transcriptase (Applied Biosystems). Semi-Quantitative PCR was conducted using specific primer pairs that amplify the fourth, fifth and sixth exons of the *white* gene (forward, 5’- CTATAGGTCATATCTTGTTTTTATTGGCAC-3’) (Reverse 3’- CGTGGGTGCCCAGTGTCC-5’) and the following parameters: denaturation 2 min at 94°C, followed by 20 cycles of 30” at 94°C, 30” at 53°C, 2’ at 72°C and final extension at 72°C for 3’. The PCR products were analyzed by agarose electrophoresis and the images were acquired and quantified with the ChemiDoc XRS imager (BioRad). The Act5C RNA was amplified as loading control with the primer pairs (forward, 5’-CCTCGTTCTTGGGAATGG-3’, Act5C reverse, 3’- CGGTGTTGGCATACAGATCCT-5’) using the same PCR cycle parameters.

### RNA-sequencing

Total long RNAs were extracted from malpighian tubuli tissues using the miRNeasy Mini Kit, following an RNeasy MinElute Cleanup Kit (Qiagen) step for the enrichment of small RNAs. The 100bp paired-end sequencing was performed at IGA using a HiSeq2000^TM^ machine. Long RNAs were mapped against the *Drosophila* genome (assembly BDGP Release 5) using TopHat (PUBMED 19289445) with default parameters. Mapped reads were associated with gene annotations (FlyBase 5.12), and transcript levels of annotated genes, expressed as Fragments per Kilobase of exon Per Million fragments mapped (FPKM), were estimated by CuffCompare (PUBMED 20436464). For small RNAs, sequencing adapters were trimmed from sequence reads with custom scripts. Resulting sequences were mapped against the *Drosophila* genome using Bowtie (PUBMED 19261174) allowing at most two substitutions within the first 20 base pairs.

### RNA-injection

*w* ^*m4h*^ *; CyORoi/Sco* 3h starved flies were allowed to lay eggs for 50 minutes on apple juice agar plates with yeast. Approximately 100–150 decorionated eggs were injected with 1–2 pl of an injection solution containing 5 mM NaCl, 5% Texas Red conjugated dextran (Molecular Probes), and 1 ng/μl of total RNA extracted and purified from testis of either Δw or *w* ^*sey*^ *Drosophila* males.

## Supporting Information

S1 Fig Screen scheme and genetic mapping. Detailed description of the cross schemes and fly genotypes used for the identification of w* alleles modifying *w* ^*m4h*^ variegation. Each of the w* allele tested (S1 Table) was screened for its ability to modify *w* ^*m4h*^ variegation when inherited from the father **(A)** or from the mother **(B)** using multiple isogenic modifier-free *w* ^*m4h*^-carrying stocks **(C, D)**. The advantage of testing w* alleles from the father is that in the F1 progeny it is possible to score not only the experimental trans-heterozygous *w* ^*m4h*^ /w* female but also the *w* ^*m4h*^ */Y* internal control males, to exclude autosomal background effects. **(E, F)** Eye pigment quantification of parental stocks (P) and the resulting trans-heterozygous females (*w* ^*m4h*^ /w*) derived from reciprocal crosses employing *w* ^*sey*^ */w* ^*sey*^ and *w* ^*1118*^ */w* ^*1118*^ females, are shown together with representative eye pictures for each genotype tested. P and F1 eye pigment quantification graphs have different scale. To map the genetic loci responsible for the increase in eye pigmentation in *w* ^*m4h*^ /w* trans-heterozygous we used classic recombination mapping on the X chromosome using recessive markers **(G)**. Single recombinant F2 males carrying the w* interacting alleles flanked by the recessive *y* ^*1*^ and cv^*1*^ markers were retested with *In(1)w* ^*m4h*^ stocks confirming that the genetic interaction scored mapped the tip of the X chromosome between cytological map 1A5 and 5A13, where the *w* gene maps in 3B6 **(H)**. To help follow the transmission of individual chromosomes in the above described crosses, female chromosomes were represented in pink while male chromosomes are shown in blue.
(JPG)

S2 Fig Hypomorphic *white* alleles increasing *w* ^*m4h*^ eye color pigmentation. Eye pigment quantification of parental stocks (P) and of the resulting trans-heterozygous female (*w* ^*m4h*^ /w*) and control male (*w* ^*m4h*^ */Y*) progeny (F1) derived from the hypomorphic *w* ^*is*^ **(A)**, *w* ^*bf*^ **(B)**, *w* ^*ch*^ **(C),** *w* ^*a4*^ **(D),** *w* ^*a*^ **(E)** and *w* ^*Bwx*^ **(F)** alleles (see S1 Table and Experimental Procedures for the exact genotypes and nature of the lesion of the alleles tested), are shown together with representative eye pictures for each genotype tested. P and F1 eye pigment quantification graphs have different scale. Note that, the parental hypomorphic w* alleles tested (P) look with darker eyes when compared to the parental *w* ^*m4h*^ line (P). However, the eye color pigment present in the population of the parental *w* ^*m4h*^ is greater than the one read from the hypomorphic w* alleles. This apparent contradiction can be explained by the fact that while the hypomorphic w* flies have all an homogenous eye color, the *w* ^*m4h*^ line eye shown in the picture is a representative example of a much more heterogeneous population containing few very dark pigmented eyes that contribute to an overall higher pigment reading.
(JPG)

S3 Fig Loss-of-function *white* alleles increasing *w* ^*m4h*^ eye color pigmentation. Eye pigment quantification of parental stocks (P) and of the resulting trans-heterozygous female (*w* ^*m4h*^ /w*) and control male (*w* ^*m4h*^ */Y*) progeny (F1) derived from the loss-of-function *w* ^*56I12*^ **(A)**, *w* ^*t*^ **(B)**, *w* ^*1*^ **(C),** *w* ^*ec3*^ **(D),** *w* ^*bf2*^ **(E)** and *w* ^*e*^ **(F)** alleles (see S1 Table and Experimental Procedures for the exact genotypes an nature of the lesion of the alleles tested), are shown together with representative eye pictures for each genotype tested. P and F1 eye pigment quantification graphs have different scale.
(JPG)

S4 Fig Cross schemes to test w* alleles effect on *E(var)*, Sb^*v*^ and *w* variegating autosomal insertions. Detailed description of cross schemes and fly genotypes tested as experimental and control classes to measure *Δw*, *w* ^*1118*^, *w* ^*sey*^ effects when in the presence of the *E(var)3-1* ^***01***^ line **(A)**, the *T(2*:*3)Sb* ^*v*^ variegating line **(B)** or finally the *39C-12*, *118E-10*, *P819*, *A4-4*, wildtype *w* autosomal variegating insertion lines (*P[w]* ^*v*^) **(C)**. All autosomal lines tested carried an X chromosome with a *w* ^*1118*^ allele. In order to clean the lines from the *w* ^*1118*^ allele, we back crossed the original lines with deletions of the *w* gene (*Df(1)w*), before testing them for their interaction with the w* suppressing alleles. To help follow the transmission of individual chromosomes, female chromosomes are represented in pink while male chromosomes are shown in blue.
(JPG)

S5 Fig Mutations in genes involved in mRNA stability do not affect *trans*-reactivation, and genomic deletions of *w* are not able to open chromatin at the *w* ^*m4h*^ locus. **(A)** Loss of function mutations in *smaug* (*smg* ^*1*^) and *pumilio* (*pum* ^*13*^), two RNA binding protein involved in mRNA destabilization in flies, are embryonic lethal. Thus, the ability of *smg* ^*1*^ and *pum* ^*13*^ loss of function alleles to modify the levels of eye pigmentation (*trans*-reactivation) we scored in *w* ^*m4h*^/w* females (*+*) was tested in heterozygosis (*pum* and *smg*). Polytene chromosome FISH using genomic probes covering the entire *w* gene (with the exception of the first intron) and coding sequences for the *hsp70* gene (mapping chromosome 3R) on homozygous *w* ^*wt*^ */w* ^*wt*^ **(B)**, and transheterozygous *w* ^*m4h*^ */Δw* **(C)** combinations. FISH signals for *white* (red) and *hsp70* (green) genomic sequences are indicated by arrowheads. The asterisk indicate the region of pericentric heterochromatin. The X indicates the chromosome where the *w* gene maps.
(JPG)

S6 Fig Cross schemes used to test *trans*-generational inheritance of *w* ^*m4h*^ *trans*-reactivation. Detailed description of cross schemes and fly genotypes tested as experimental and control classes to measure *trans*-generational inheritance of *w* ^*m4h*^ *trans*-reactivation from (F1) trans-heterozygous *w* ^*m4h*^/w* females when crossed with parental w*/Y (**A**) or *Δw /Y* (**B**) males. To help follow the transmission of individual chromosomes, female chromosomes are represented in pink while male chromosomes are shown in blue. (**C**) *Trans*-generational inability of a wild type *w* ^*+*^ allele (coming from an *OreR* stock) to *trans*-reactivate in F2 the *w* ^*m4h*^ locus. Eye pigment quantification and representative eye pictures of the parental (P), F1 and F2 progenies for each genotype tested are shown.
(JPG)

S7 Fig Cross schemes used to test that *trans*-reactivation is mediated by a diffusible factor or influenced by mutations in PTGS genes. Detailed description of cross schemes and fly genotypes tested as experimental **(A)** and control **(B)** classes to measure *trans*-reactivation of *w* ^*m4h*^ during the process of gynogenesis. **(C)** Cross scheme used to test in heterozygosis the ability of loss of function alleles (Mut) to modify *trans*-reactivation. **(D)** Eye pigment quantification was conducted on adult flies of the indicated genotype and the Log_2_ ratio of *w* ^*m4h*^/w* trans-reactivation in the mutant background over the normal levels of *w* ^*m4h*^/w* trans-reactivation (normalized for the respective readings on the *w* ^*m4h*^/*w* ^*m4h*^ lines alone) was used as a measure of the influence of the mutation tested in the onset of *trans*-reactivation. To help follow the transmission of individual chromosomes, female chromosomes are represented in pink while male chromosomes are shown in blue.
(JPG)

S1 Table Genotypes and nature of the lesion of the w* alleles tested. Genotypes of stocks carrying the w* alleles tested for their ability to modify in trans-heterozygosity the *w* ^*m4h*^ eye color variegation. Stocks that increased eye color variegation are highlighted in orange (Suppressors), the ones that did not have any effect are highlighted in light red (-). Tested *white* genomic deletions are highlighted in light grey. Finally, the mutation class, the mutagen employed and the Nature of the lesion, for each w* allele tested is reported when known.
(DOCX)
