# Supplementary figures and images for: Trans-Reactivation: A New Epigenetic Phenomenon Underlying Transcriptional Reactivation of Silenced Genes
Source: PLoS Genet. 2015 Aug 20;11(8):e1005444. doi: 10.1371/journal.pgen.1005444 (PMC4546373; doi:10.1371/journal.pgen.1005444)

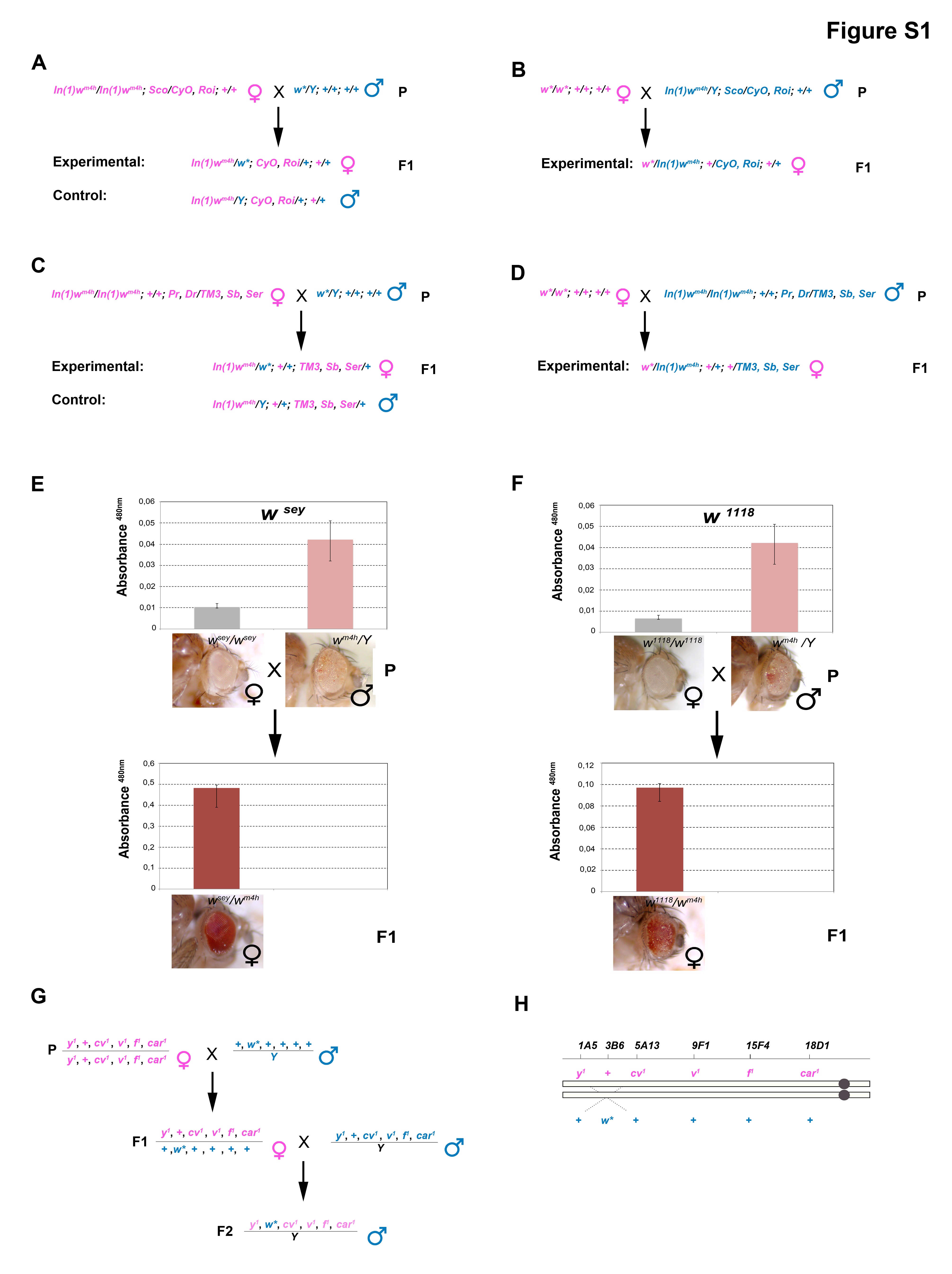

Supplement: S1 Fig — Detailed description of the cross schemes and fly genotypes used for the identification of w* alleles modifying w m4h variegation. Each of the w* allele tested (S1 Table) was screened for its ability to modify w m4h variegation when inherited from the father (A) or from the mother (B) using multiple isogenic modifier-free w m4h-carrying stocks (C, D). The advantage of testing w* alleles from the father is that in the F1 progeny it is possible to score not only the experimental trans-heterozygous w m4h /w* female but also the w m4h /Y internal control males, to exclude autosomal background effects. (E, F) Eye pigment quantification of parental stocks (P) and the resulting trans-heterozygous females (w m4h /w*) derived from reciprocal crosses employing w sey /w sey and w 1118 /w 1118 females, are shown together with representative eye pictures for each genotype tested. P and F1 eye pigment quantification graphs have different scale. To map the genetic loci responsible for the increase in eye pigmentation in w m4h /w* trans-heterozygous we used classic recombination mapping on the X chromosome using recessive markers (G). Single recombinant F2 males carrying the w* interacting alleles flanked by the recessive y 1 and cv1 markers were retested with In(1)w m4h stocks confirming that the genetic interaction scored mapped the tip of the X chromosome between cytological map 1A5 and 5A13, where the w gene maps in 3B6 (H). To help follow the transmission of individual chromosomes in the above described crosses, female chromosomes were represented in pink while male chromosomes are shown in blue. (JPG) [file pgen.1005444.s001.jpg]

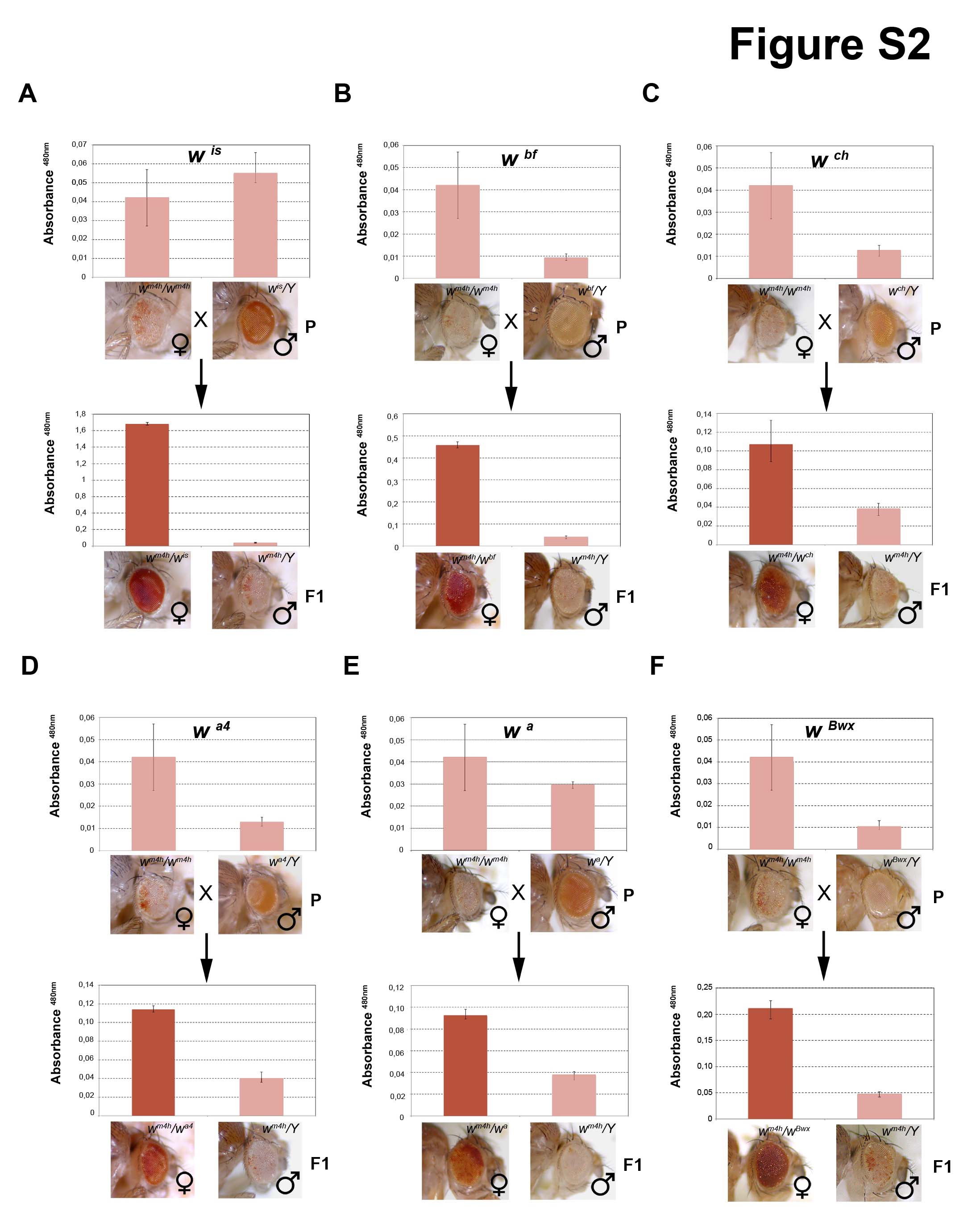

Supplement: S2 Fig — Eye pigment quantification of parental stocks (P) and of the resulting trans-heterozygous female (w m4h /w*) and control male (w m4h /Y) progeny (F1) derived from the hypomorphic w is (A), w bf (B), w ch (C), w a4 (D), w a (E) and w Bwx (F) alleles (see S1 Table and Experimental Procedures for the exact genotypes and nature of the lesion of the alleles tested), are shown together with representative eye pictures for each genotype tested. P and F1 eye pigment quantification graphs have different scale. Note that, the parental hypomorphic w* alleles tested (P) look with darker eyes when compared to the parental w m4h line (P). However, the eye color pigment present in the population of the parental w m4h is greater than the one read from the hypomorphic w* alleles. This apparent contradiction can be explained by the fact that while the hypomorphic w* flies have all an homogenous eye color, the w m4h line eye shown in the picture is a representative example of a much more heterogeneous population containing few very dark pigmented eyes that contribute to an overall higher pigment reading. (JPG) [file pgen.1005444.s002.jpg]

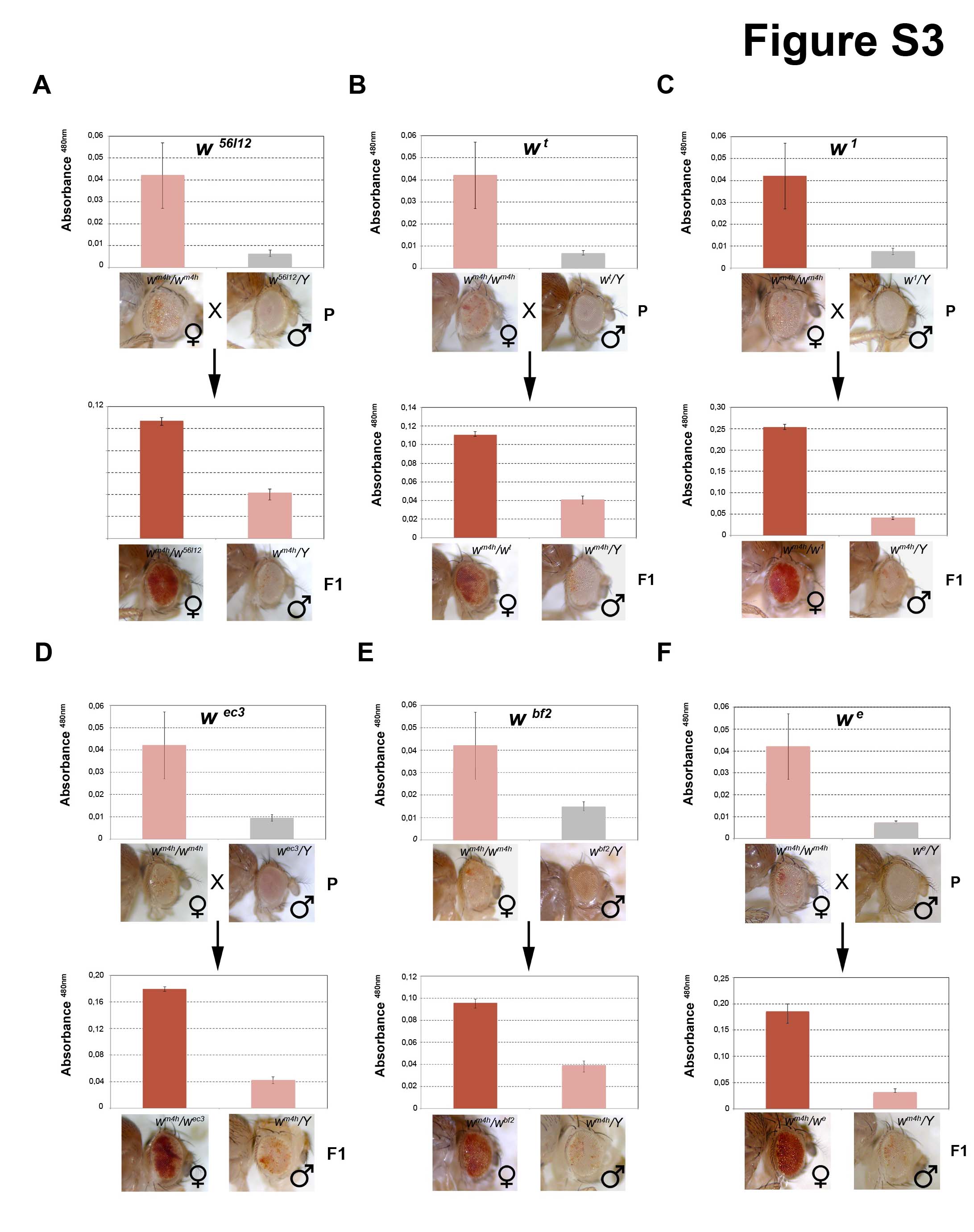

Supplement: S3 Fig — Eye pigment quantification of parental stocks (P) and of the resulting trans-heterozygous female (w m4h /w*) and control male (w m4h /Y) progeny (F1) derived from the loss-of-function w 56I12 (A), w t (B), w 1 (C), w ec3 (D), w bf2 (E) and w e (F) alleles (see S1 Table and Experimental Procedures for the exact genotypes an nature of the lesion of the alleles tested), are shown together with representative eye pictures for each genotype tested. P and F1 eye pigment quantification graphs have different scale. (JPG) [file pgen.1005444.s003.jpg]

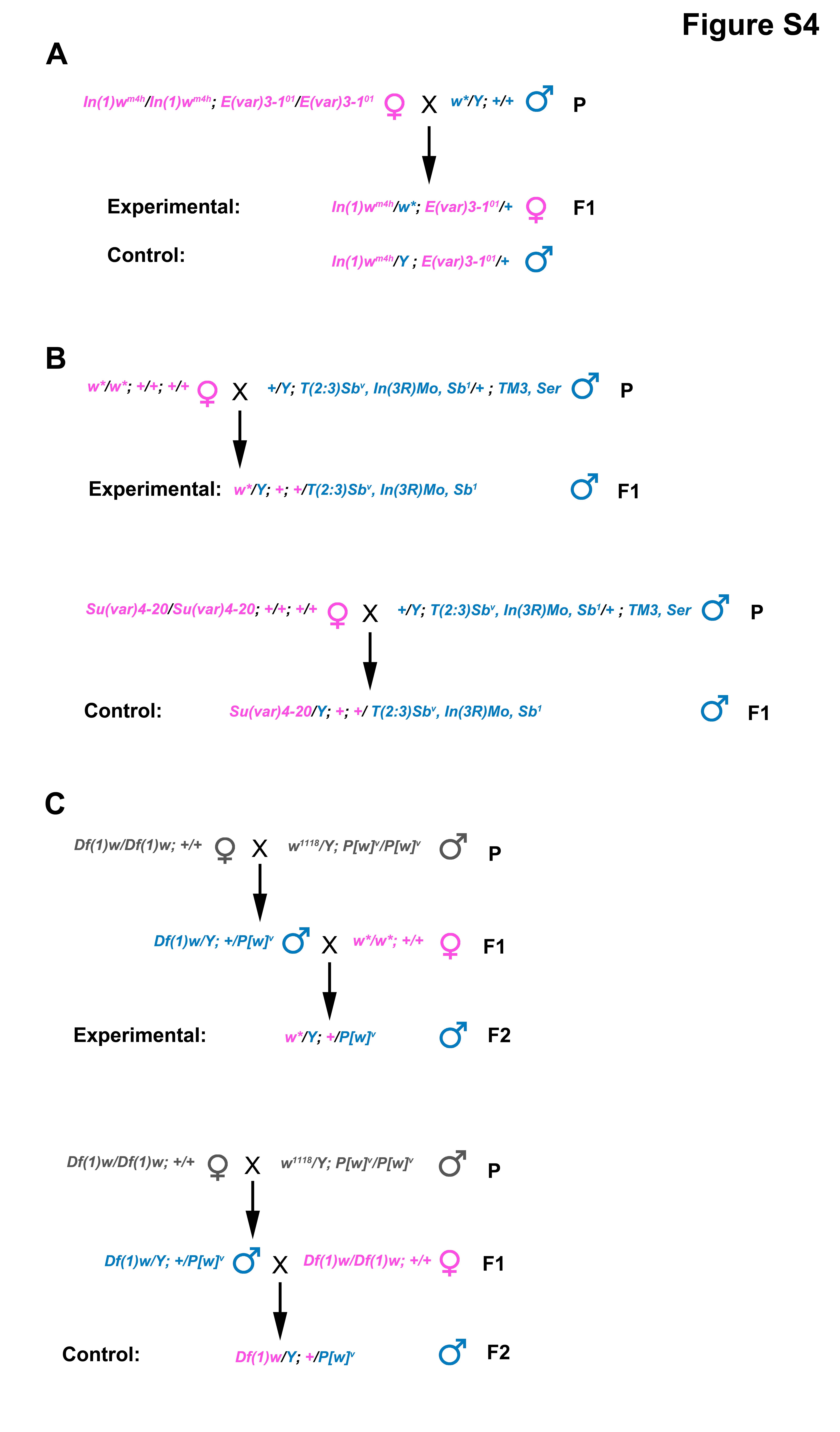

Supplement: S4 Fig — Detailed description of cross schemes and fly genotypes tested as experimental and control classes to measure Δw, w 1118, w sey effects when in the presence of the E(var)3-1 01 line (A), the T(2:3)Sb v variegating line (B) or finally the 39C-12, 118E-10, P819, A4-4, wildtype w autosomal variegating insertion lines (P[w] v) (C). All autosomal lines tested carried an X chromosome with a w 1118 allele. In order to clean the lines from the w 1118 allele, we back crossed the original lines with deletions of the w gene (Df(1)w), before testing them for their interaction with the w* suppressing alleles. To help follow the transmission of individual chromosomes, female chromosomes are represented in pink while male chromosomes are shown in blue. (JPG) [file pgen.1005444.s004.jpg]

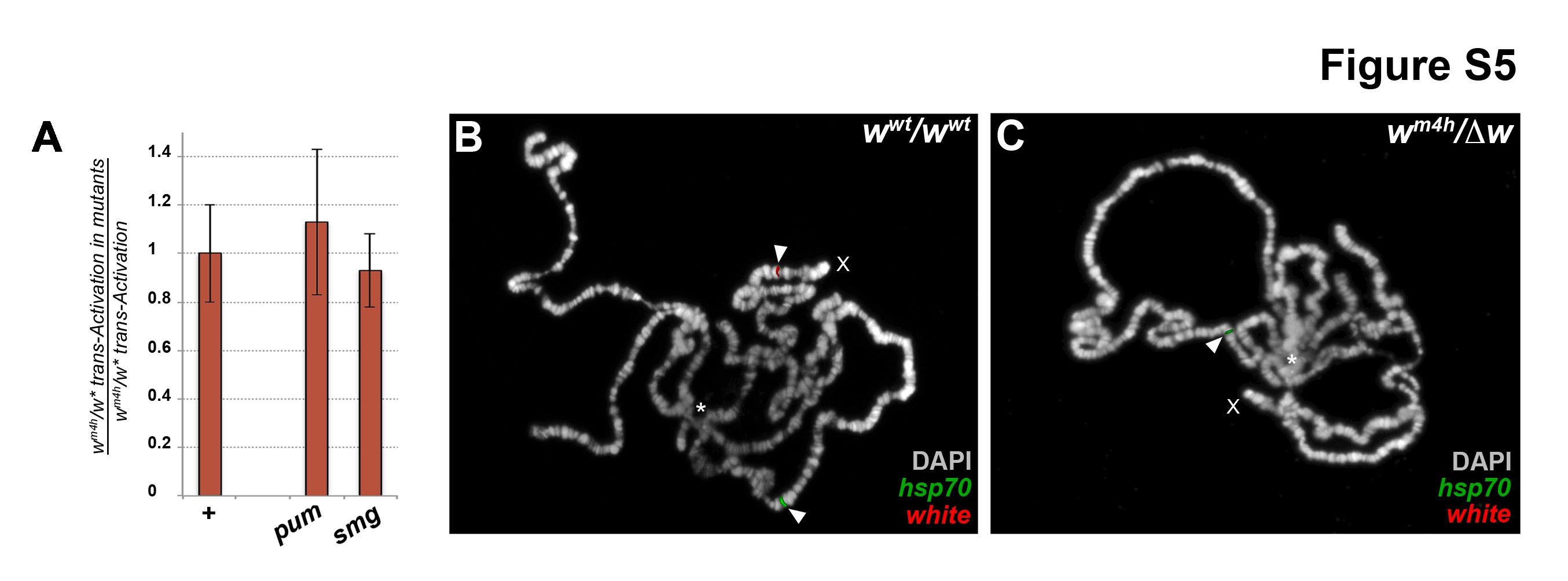

Supplement: S5 Fig — (A) Loss of function mutations in smaug (smg 1) and pumilio (pum 13), two RNA binding protein involved in mRNA destabilization in flies, are embryonic lethal. Thus, the ability of smg 1 and pum 13 loss of function alleles to modify the levels of eye pigmentation (trans-reactivation) we scored in w m4h/w* females (+) was tested in heterozygosis (pum and smg). Polytene chromosome FISH using genomic probes covering the entire w gene (with the exception of the first intron) and coding sequences for the hsp70 gene (mapping chromosome 3R) on homozygous w wt /w wt (B), and transheterozygous w m4h /Δw (C) combinations. FISH signals for white (red) and hsp70 (green) genomic sequences are indicated by arrowheads. The asterisk indicate the region of pericentric heterochromatin. The X indicates the chromosome where the w gene maps. (JPG) [file pgen.1005444.s005.jpg]

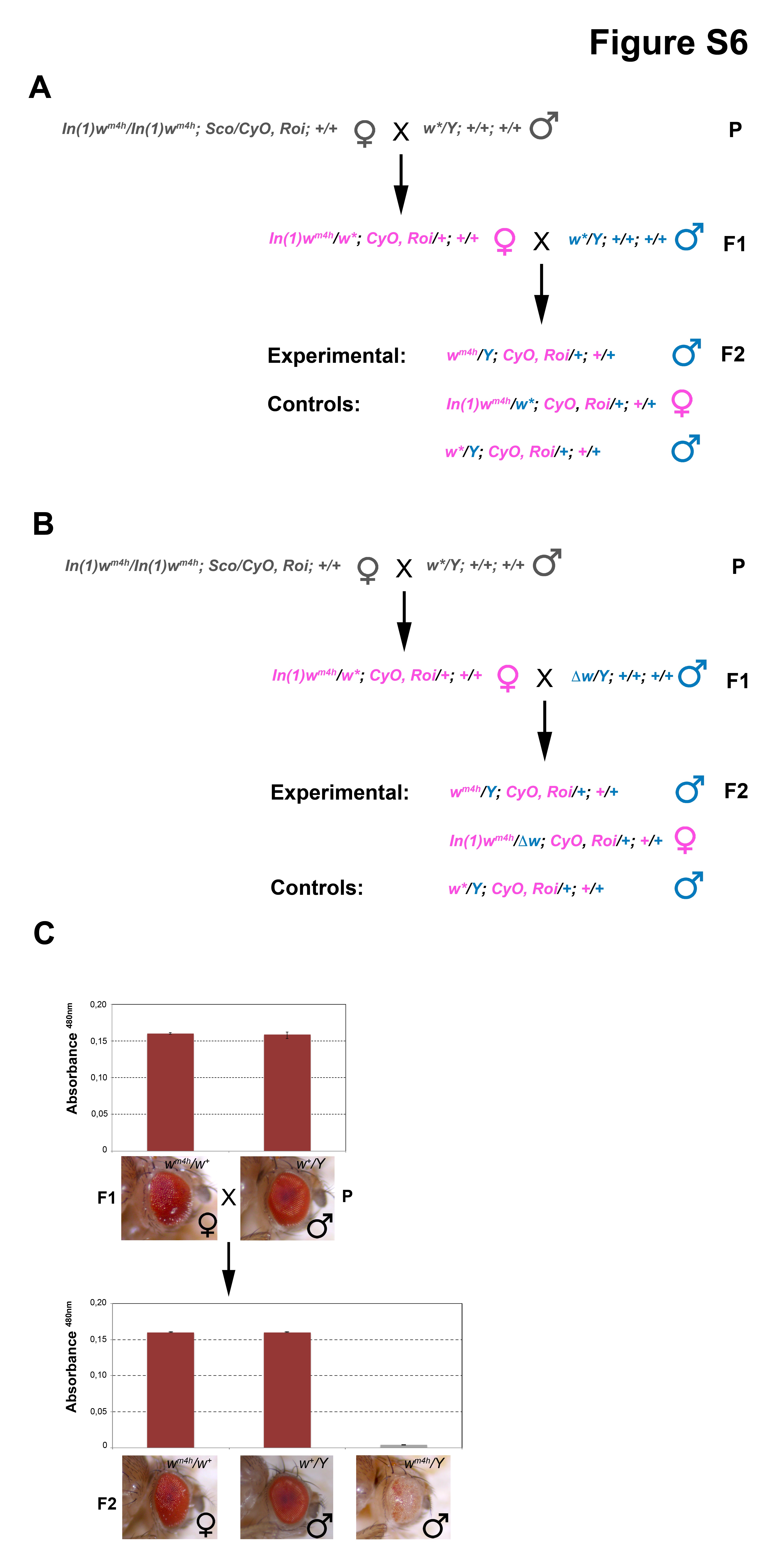

Supplement: S6 Fig — Detailed description of cross schemes and fly genotypes tested as experimental and control classes to measure trans-generational inheritance of w m4h trans-reactivation from (F1) trans-heterozygous w m4h/w* females when crossed with parental w*/Y (A) or Δw /Y (B) males. To help follow the transmission of individual chromosomes, female chromosomes are represented in pink while male chromosomes are shown in blue. (C) Trans-generational inability of a wild type w + allele (coming from an OreR stock) to trans-reactivate in F2 the w m4h locus. Eye pigment quantification and representative eye pictures of the parental (P), F1 and F2 progenies for each genotype tested are shown. (JPG) [file pgen.1005444.s006.jpg]
