# Supplementary material for: Trans-Reactivation: A New Epigenetic Phenomenon Underlying Transcriptional Reactivation of Silenced Genes
Source: PLoS Genet. 2015 Aug 20;11(8):e1005444. doi: 10.1371/journal.pgen.1005444 (PMC4546373; doi:10.1371/journal.pgen.1005444)
Supplement: S1 Table — Genotypes of stocks carrying the w* alleles tested for their ability to modify in trans-heterozygosity the w m4h eye color variegation. Stocks that increased eye color variegation are highlighted in orange (Suppressors), the ones that did not have any effect are highlighted in light red (-). Tested white genomic deletions are highlighted in light grey. Finally, the mutation class, the mutagen employed and the Nature of the lesion, for each w* allele tested is reported when known. (DOCX) [file pgen.1005444.s008.docx]

| **Genotypes of *w** Alleles Tested** | **Interaction with *wm4h*** | **Class** | **Mutagen** | **Nature of the lesion** | **Stock#** |
| --- | --- | --- | --- | --- | --- |
| ***w[a]*** | Suppressor | Hypomorphyic allele | Spontaneus | copia insertion in the second intron | BL148 |
| ***w[a4]*** | Suppressor | - | Spontaneus | Insertion of a 3S18 element into the second intron | BL152 |
| ***w[bf2****]* | Suppressor | - | X ray | Insertion of a roo element (in antiparallel orientation) into the 5' untranslated region | BL154 |
| ***w[bf]*** *f[5]* | Suppressor | Hypomorphic allele | Spontaneus | insertion of a roo element in 4 Intron | BL157 |
| ***w[ec3]*** | Suppressor | - | X ray | Unknown | BL161 |
| ***w[t]*** *fw[1]* | Suppressor | Hypomorphic allele | Spontaneus | Unknown | BL166 |
| ***w[1]*** | Suppressor | Loss of function allele | Spontaneus | Insertion of 4.9kb Doc element, close to the site of transcription initiation | BL2390 |
| ***w[ch]*** *wy[1]* | Suppressor | Hypomorphic allele | Spontaneus | Pogo insertion into Doc of w1 (close to the site of transcription initiation) | BL4451 |
| ***w[sey]****; se[1]* | Suppressor | - | Spontaneus |  | BL4474 |
| ***w[1118]*** | Suppressor | Loss of function allele | Spontaneus | Partial deletion of w 5' to site of insertion of P-element in whd80k17, including exon 1 | BL3605 |
| *br1* ***w[e]*** *ec1 rb1 t4* | Suppressor | Hypomorphic allele | Spontaneus | pogo reversion of w1 | BL14 |
| *z1* ***w[Bwx]*** *t1 v1 m74f* | Suppressor | - | Spontaneus | Point mutation | BL1293 |
| *pn[1] z[1]* ***w[is]*** | Suppressor | - | Spontaneus | - | Kyoto 101-168 |
| ***w[56l12]*** | Suppressor | - | - | - | Kyoto 101-204 |
| *z[1]* ***w[11E4]*** | **-** | - | X ray | Deletion of the entire locus | BL200 |
| ***w[h]*** | **-** | Hypomorphic allele | Spontaneus | Insertion of a roo element into the Doc element present in w1 | BL162 |
| ***w[118]****; Df(2R)H3C1/CyO* | **-** | - | EMS | There is no detectable alteration in the restriction enzyme profile compared to wild-type | BL198 |
| *y[1]* ***w[i]*** | **-** | Hypomorphic allele | Spontaneus | Progenitor w[1] (Duplication of sequences from intron 1 to the start of exon 3) | Kyoto 101-254 |
| ***w[sp55****]* | **-** | - | Spontaneus | Insertion of a mdg3 element into the 5' untranslated region | Kyoto 101-233 |
| ***Df(1)w258-42****, y[1]* | **-** | Deletion | - | 3A5--3C5 | BL733 |
| ***Df(1)w258-45****, y[1]* | **-** | Deletion | - | 3B3--3C2 | BL734 |
| ***Df(1)w[67c23]*** | **-** | Deletion | - | 3B6-3C2 | BL6599 |
